# Spreading of Heterochromatin Is Limited to Specific Families of Maize Retrotransposons

**DOI:** 10.1371/journal.pgen.1003127

**Published:** 2012-12-13

**Authors:** Steven R. Eichten, Nathanael A. Ellis, Irina Makarevitch, Cheng-Ting Yeh, Jonathan I. Gent, Lin Guo, Karen M. McGinnis, Xiaoyu Zhang, Patrick S. Schnable, Matthew W. Vaughn, R. Kelly Dawe, Nathan M. Springer

**Affiliations:** 1 Microbial and Plant Genomics Institute, Department of Plant Biology, University of Minnesota, Saint Paul, Minnesota, United States of America; 2 Department of Plant Biology, University of Georgia, Athens, Georgia, United States of America; 3 Biology Department, Hamline University, Saint Paul, Minnesota, United States of America; 4 Center for Plant Genomics and Department of Agronomy, Iowa State University, Ames, Iowa, United States of America; 5 Department of Biological Science, Florida State University, Tallahassee, Florida, United States of America; 6 Texas Advanced Computing Center, University of Texas at Austin, Austin, Texas, United States of America; University of California Irvine, United States of America

## Abstract

Transposable elements (TEs) have the potential to act as controlling elements to influence the expression of genes and are often subject to heterochromatic silencing. The current paradigm suggests that heterochromatic silencing can spread beyond the borders of TEs and influence the chromatin state of neighboring low-copy sequences. This would allow TEs to condition obligatory or facilitated epialleles and act as controlling elements. The maize genome contains numerous families of class I TEs (retrotransposons) that are present in moderate to high copy numbers, and many are found in regions near genes, which provides an opportunity to test whether the spreading of heterochromatin from retrotransposons is prevalent. We have investigated the extent of heterochromatin spreading into DNA flanking each family of retrotransposons by profiling DNA methylation and di-methylation of lysine 9 of histone 3 (H3K9me2) in low-copy regions of the maize genome. The effects of different retrotransposon families on local chromatin are highly variable. Some retrotransposon families exhibit enrichment of heterochromatic marks within 800–1,200 base pairs of insertion sites, while other families exhibit very little evidence for the spreading of heterochromatic marks. The analysis of chromatin state in genotypes that lack specific insertions suggests that the heterochromatin in low-copy DNA flanking retrotransposons often results from the spreading of silencing marks rather than insertion-site preferences. Genes located near TEs that exhibit spreading of heterochromatin tend to be expressed at lower levels than other genes. Our findings suggest that a subset of retrotransposon families may act as controlling elements influencing neighboring sequences, while the majority of retrotransposons have little effect on flanking sequences.

## Author Summary

Transposable elements comprise a substantial portion of many eukaryotic genomes. These mobile fragments of DNA can directly mutate genes through insertions into coding regions but may also affect the gene regulation through nearby insertions. There is evidence that the majority of transposable elements are epigenetically silenced, and in some cases this silencing may spread to neighboring sequences. This spreading of heterochromatin could create a significant fitness tradeoff between transposon silencing and gene expression. The maize genome has a complex organization with many genes flanked by retrotransposons, providing an opportunity to study the interaction of retrotransposons and genes. To survey the prevalence of heterochromatin spreading associated with different retrotransposon families, we profiled the spread of heterochromatin into nearby low copy sequences for 150 high copy retrotransposon families. While many retrotransposons exhibit little to no spreading of heterochromatin, there are some retrotransposon families that do exhibit spreading. Genes located near retrotransposons that spread heterochromatin have lower expression levels. The families of retrotransposons that spread heterochromatin marks to nearby low-copy sequences may have increased fitness costs for the host genome due to their suppression of genes located near insertions.

## Introduction

A substantial fraction of most eukaryotic genomes is composed of transposable elements (TEs) [1]–[4]. While these TEs are sometimes referred to as “junk” DNA, there is evidence for potential functional roles in some instances [5]. Indeed, Barbara McClintock used the term “controlling elements” to describe the potential for these sequences to affect the regulation of endogenous genes [6]–[7]. Mobile genetic elements include class I retrotransposons and class II DNA transposons [2]. The class I TEs transpose via an RNA intermediate while class II TEs utilize a DNA intermediate for transposition. There are a variety of sub-families of both types of TEs [2] that differ in structure, activity, and integration patterns.

TEs could influence neighboring genes by providing regulatory elements or promoters that would alter expression levels or patterns [8]–[9]. Alternatively, TEs may be targeted for silencing and this silencing could spread to affect neighboring sequences potentially including endogenous genes or regulatory elements [10]–[12]. There are several examples in which heterochromatic silencing of TEs can influence expression of nearby genes, including the *agouti* and *Axin* locus in mouse [13]–[15], *FLC* [16], *FWA* [17] and BNS [18] in Arabidopsis and sex-determination in melons [19]. While there are examples of heterochromatin spreading from retrotransposons to neighboring sequences, it is unclear how general this phenomenon is. Whole genome profiling of DNA methylation in Arabidopsis [20] found that the level of DNA methylation often had sharp boundaries at the edge of repeats although some inverted repeats did exhibit spreading. Another study [21] found limited (200–500 bp) spreading of DNA methylation surrounding some TEs in Arabidopsis. There is evidence that highly methylated TEs are under-represented near genes in Arabidopsis and it has been suggested that the silencing of TEs located near genes might have deleterious consequences [21]–[23]. There is evidence for variation in the spreading of heterochromatin for different families of TEs in mouse [24] and evidence that differences in TE insertions contribute to gene expression variation in other rodents [25].

The complex organization of the maize genome, with interspersed genes and TEs [26]–[28], provides an excellent system in which to study the effects of retrotransposons on neighboring DNA. Many model organisms have relatively small, compact genomes with relatively few retrotransposons. Since these genomes do not have a number of moderate-high copy retrotransposon families it can be difficult to assess the variation in spreading of heterochromatin to neighboring low-copy sequences. The maize genome is more representative of the organization of sequences observed within most flowering plants and is similar to the organization of many mammalian genomes as well. There are a large number of distinct families of retrotransposons within the maize genome and many of these families are moderate to high copy number [28]–[31]. In addition, haplotypes differ substantially with regard to the presence or absence of specific retrotransposon insertions [31]–[34]. The majority of repetitive sequences, including retrotransposons, in the maize genome are highly methylated [26], [35]–[38].

The existence of heavily silenced retrotransposons interspersed with genes throughout the maize genome provides ample opportunities for TEs to exert epigenetic regulation on surrounding sequences. We were interested in further documenting the extent of heterochromatin spreading from maize retrotransposons to neighboring sequencings. Genomic profiling of DNA methylation and H3K9me2 found that heterochromatic spreading is only observed for a small number of specific retrotransposon families. These families tend to be enriched in pericentromeric regions of chromosomes. The analysis of haplotypes lacking specific retrotransposon insertions provides evidence that the adjacent heterochromatin is the result of spreading rather than insertion site bias.

## Results

### Heterochromatin spreads from some retrotransposons within the maize genome

DNA methylation and chromatin modifications were profiled for low-copy sequences in the maize genome using methylated DNA immunoprecipitation (meDIP) and chromatin-immunoprecipitation (ChIP) with antibodies specific for H3K9me2 or H3K27me3, respectively. The fractions of the genome enriched for DNA or histone modifications were hybridized to a high-density microarray containing ∼2.1 million long oligonucleotide probes derived from the unmasked, non-repetitive fraction of the maize genome. The probes are spaced every 200 bp in the low-copy portions of the maize genome and can provide a profile for the chromatin state in these regions [39]. Our analyses focused on a subset of ∼1.4 million probes that are single-copy (no other sequences with at least 90% identity within maize genome sequence). While this approach does not provide information on the chromatin state within repetitive sequences it can assess how retrotransposons impact neighboring sequences [39]. An independent whole-genome bisulphite sequencing dataset (∼7× coverage) was used to further confirm the patterns that we observed in the meDIP-chip experiments. This independent approach was able to assess DNA methylation within retrotransposons as well as low-copy sequences. The enrichment for sequences associated with H3K9me2 was validated using a set of known sequences (Figure S1A) and several sequences identified by the profiling experiments (Figure S1B).

**Figure 1:**
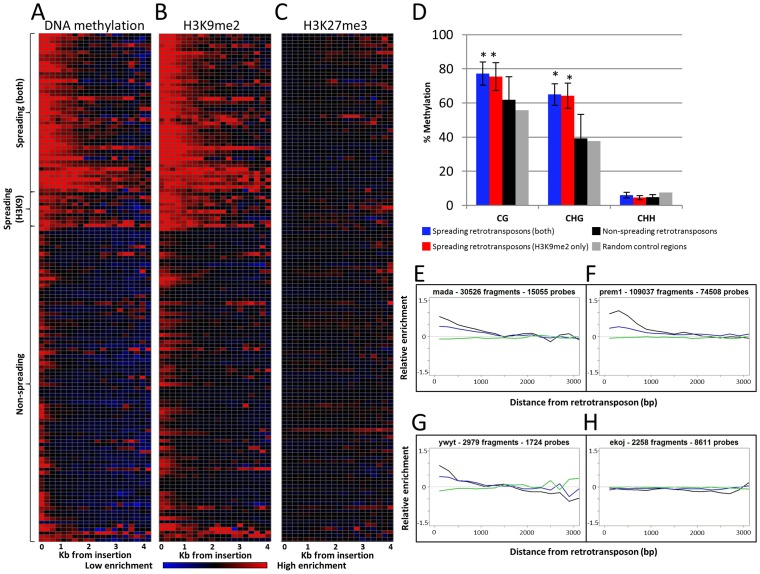
Heterochromatin spreading is restricted to some retrotransposon families. The 144 families of class I retrotransposons represented by at least 1000 probes within the adjacent 4 kb were identified and the average level of H3K9me2, 5-methylcytosine and K3K27me3 enrichment was determined for 200 bp bins. The average values are calculated from both sides of annotated elements collapsed. (A) The relative level of DNA methylation in each 200 bp bin is shown for each of the 144 families. The red color indicates enrichment for the modification while blue indicates depletion of the mark. Black indicates levels of the modification similar to genome-wide average values. The color intensity is based on the average log ratio of immunoprecipitated DNA relative to input DNA. The retrotransposons are grouped according to whether they show spreading for DNA methylation and H3K9me2, H3K9me2 only or neither of the marks. Similar plots are shown for H3K9me2 (B) and H3K27me3 (C). (D) Whole-genome bisulphite sequencing data was used to assess the methylation level in different cytosine contexts in the low-copy (based on the absence of repetitive sequences) 1 kilobase regions flanking spreading (both), spreading (H3K9), non-spreading retrotransposons and for 10,000 random genomic regions. The error bars indicate standard deviation among retrotransposon families and “*” indicate significant (p<0.001) differences for a group relative to the non-spreading families. The level of CG and CHG methylation is higher for spreading retrotransposons than for non-spreading retrotransposons. (E–H) show plots of representative family(s) for each class. The relative abundance of DNA methylation (black), H3K9me2 (blue) and H3K27me3 (green) is shown for the first 3 kb of low-copy DNA flanking the retrotransposon family on both sides. The y-axis indicates enrichment relative to genome-wide average (value of 0 is equal to average of permuted data).

A large number of class I TEs (retrotransposons) have been identified within the maize genome [30]. These retrotransposons tend to be highly methylated in CG and CHG sequence contexts (Figure S2). We assessed whether heterochromatic chromatin modifications would be enriched in the single-copy regions that flank these retrotransposons. The chromatin state of sequences adjacent to any specific insertion of a retrotransposon is influenced by regulatory and insulator sequences as well as any potential effects of nearby retrotransposons. By assessing the average level of chromatin modifications near all of the retrotransposons of the same family it is possible to identify whether retrotransposon families vary in their influence on local chromatin state. Single-copy probes that are located within 4 kb of all retrotransposons were identified and used to assess the level of chromatin modifications in 200 bp bins of low-copy sequences adjacent to superfamilies, such as gypsy or copia (Figure S3) and individual families of retrotransposons (Figure 1). Many of the retrotransposon families exhibit elevated levels of DNA methylation and H3K9me2 in the 200 bp immediately adjacent to their insertion sites (Figure S3). Because the meDIP-chip profiling of DNA methylation has a resolution of 300–500 bp it is likely that some of the apparent increase in DNA methylation levels very close to retrotransposons represents DNA methylation within the repeats themselves.

A subset of the retrotransposon families also exhibit elevated levels of DNA methylation and H3K9me2 in regions more than 200 bp away from their insertion sites. In general, levels of H3K9me2 and DNA methylation were well correlated, but there were some families with different enrichment for these two marks. As expected, there was no evidence for enrichment (or depletion) of the facultative heterochromatin mark, H3K27me3, in regions flanking the retrotransposons (Figure 1C). To identify retrotransposon families associated with significant levels of spreading of heterochromatic chromatin modifications in adjacent low-copy sequences we compared the distribution of methylation levels in each 200 bp bin with a set of randomly permuted data (10,000 randomly assigned “insertions”) and defined whether each 200 bp bin had significantly higher levels of a chromatin modification than random genomic sequences. Retrotransposon families that exhibit significant (p<0.001) enrichment for a chromatin modification for each bin up through at least 800 bp were classified as spreading families. There are 39 retrotransposon families that exhibit significant enrichments of DNA methylation and H3K9me2 within each of the first four 200 bp bins adjacent to their insertion sites. These families will hereafter classified as “spreading (both)” families (Figure 1A–1B, 1E–1F and Figure S4). Another 10 retrotransposon families had significant levels of H3K9me2 but did not have at least 800 bp of significant enrichment for DNA methylation. These families will hereafter be classified as “spreading (H3K9)” (Table S1; Figure 1A–1B, 1G and Figure S5). Many of these H3K9 only spreading families have elevated levels of DNA methylation in these same regions (Figure S4), but do not pass the significance threshold for all bins within the adjacent 800 base pairs. The remaining 95 retrotransposon families did not exhibit significant enrichment for either DNA methylation or H3K9me2 (example in Figure 1H). There was no evidence for significant enrichment of H3K27me3 in regions near any retrotransposon families (Figure 1C). The initial classification of retrotransposon families was based upon chromatin profiles from B73 seedling tissue. However, very similar patterns were observed for other genotypes and tissues. Specifically, the same families have significant enrichments of DNA methylation in Mo17 seedling, B73 endosperm and B73 embryo tissue (Figure S6). The H3K9me2 patterns are quite similar in both B73 and Mo17 seedlings (Figure S7A–S7B) and there was no evidence for enrichment for H3K27me3 in any of the tissues or genotypes assessed (Figure S7C–S7E).

The analysis of the whole-genome bisulphite sequencing data supports the classifications of different retrotransposon families (Figure 1D and Figure S2). Both CG and CHG DNA methylation levels are higher in low-copy regions flanking spreading (both) and spreading (H3K9) families (Figure 1D). The level of DNA methylation is higher in sequences flanking spreading (both) retrotransposon families than for sequences flanking spreading (H3K9) retrotransposons. The sequences flanking the non-spreading families have DNA methylation levels that are similar to randomly selected genomic regions (Figure 1D). The analysis of internal (within the repeat itself) DNA methylation levels (Figure S2) reveals that the levels of CG methylation within retrotransposons with, or without spreading are similar. However, the spreading (both) and spreading (H3K9) retrotransposon families have slightly elevated levels of CHG methylation at internal sequences. Interestingly, the non-spreading retrotransposon families tend to have higher levels of internal CHH methylation than do spreading families (Figure S2). The relative levels of H3K9me2 within retrotransposons was assessed by qPCR for 10 of the families, including six spreading (both) and four non-spreading families (Figure S8). There was no evidence for higher levels of H3K9me2 within the families that exhibit heterochromatic spreading than for those that do not (Figure S8). The elevated levels of DNA methylation and/or H3K9me2 in low copy sequences flanking the insertion sites observed for a subset of the retrotransposon families are largely confined to the region within 800–1,600 bp of the insertion site (Figure 1A–1B). A closer examination of the levels of DNA methylation and H3K9me2 near each spreading family indicates a fairly sharp drop to non-significant levels of the modifications within 2 kb of the insertion site (Figure 1E–1G; Figures S4, S5) for spreading families. The visualization of individual spreading families (Figures S4, S5) reveals that the distance of heterochromatin spreading varies for different retrotransposon families. This analysis provides clear evidence for diversity in the prevalence of heterochromatin found in low-copy regions flanking different families of retrotransposons in the maize genome.

### Spreading of heterochromatin does not require CMT or Mop1

**Figure 2:**
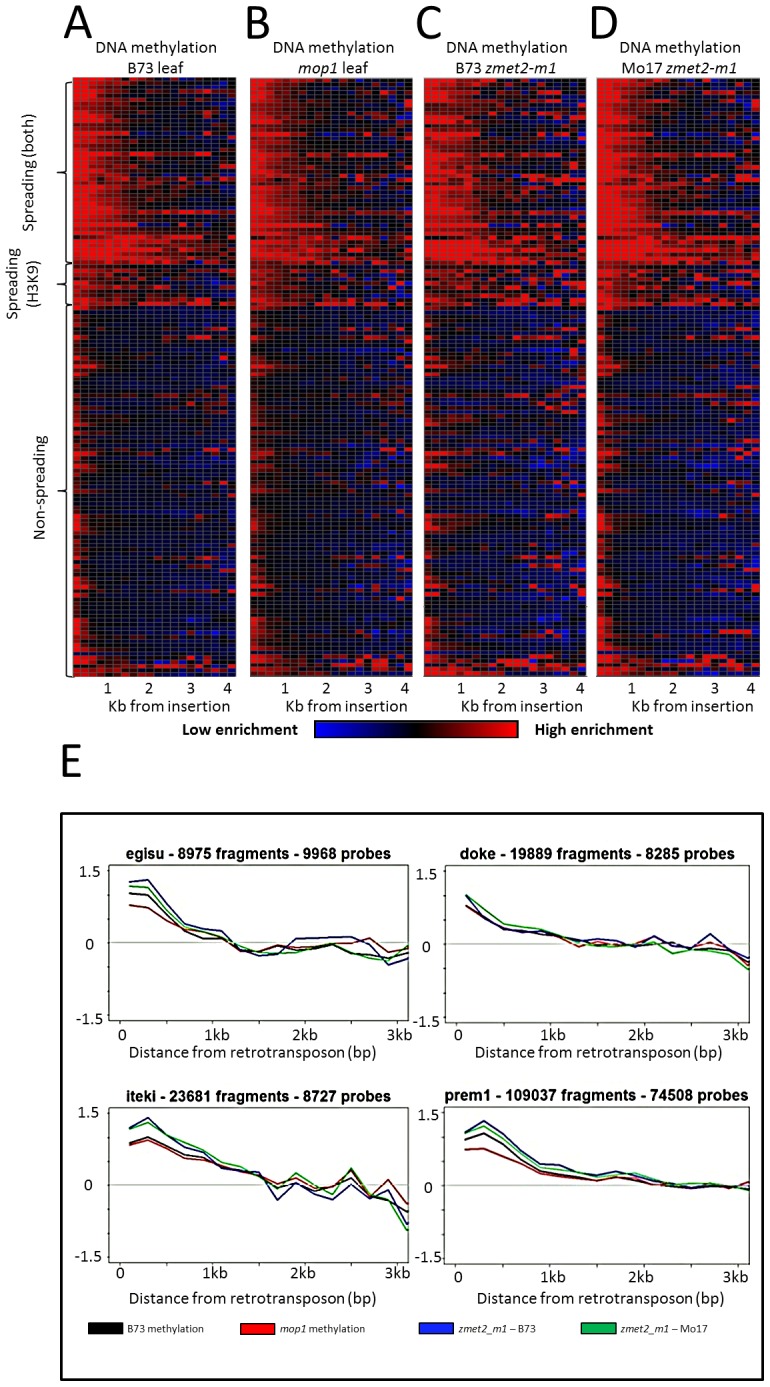
DNA methylation enrichment near retrotransposons is not affected by mop1 or zmet2-m1 mutations. The relative level of DNA methylation in each 200 bp bin is shown for each of the 144 retrotransposon families. Modification levels calculated include probes flanking both ends of retrotransposns. The red color indicates enrichment for the modification while blue indicates depletion of the mark. Black indicates levels of the modification similar to genome-wide average values. The retrotransposons are grouped according to whether they show spreading for DNA methylation and H3K9me2, just H3K9me2 or neither of the marks. The profiles are shown for B73 leaf (A), homozygous *mop1* mutant (B), homozygous mutant *zmet2-m1* in B73 (C) and Mo17 (D) genetic backgrounds. Example plots of methylation levels in the same backgrounds (E) indicate minimal differences between mutant backgrounds on spreading.

The mechanistic basis for the spreading of heterochromatin is not well defined. It is possible that the interplay between DNA methylation and histone modifications [40]–[41] would result in spreading of chromatin modifications beyond the specific target. To probe the mechanistic basis of spreading we profiled DNA methylation levels in several maize mutants that are known, or expected, to affect DNA methylation patterns. In plants, one pathway that impacts DNA methylation is RNA-directed, and requires the activity of multiple RNA polymerases (RNA PolIV and PolV), an RNA dependent RNA polymerase (RDR2), a dicer like protein, and multiple chromatin modifiers [42]. The *mop1* mutant of the maize *Rdr2* gene [43]–[45] exhibits variable expression of specific retrotransposon families in mutant relative to wild-type tissue [46]. However, we found no evidence for a consistent effect of the *mop1* mutation on the expression levels of spreading or non-spreading retrotransposon families. Indeed, spreading retrotransposon families include examples of both up- and down-regulation in mop1 mutant individuals relative to wild-type (Table S1). In addition, there were examples of non-spreading retrotransposon families that do, and do not, exhibit altered expression in mop1 plants. The levels of DNA methylation in low-copy sequences neighboring retrotransposon families was analyzed in the *mop1* mutant to assess whether the spreading of heterochromatin might be affected (Figure 2). There was no evidence for a reduction in the distance or magnitude of the spreading of DNA methylation in the *mop1* mutants relative to wild-type plants. The small RNA profile of spreading and non-spreading retrotransposon families was assessed using a recently published small RNA profile based on B73 shoot tissue [47]. The average count of small RNAs per retrotransposon and coverage of retrotransposon did not vary between spreading (both), spreading (H3K9) or non-spreading retrotransposon families (Figure S9).

Spreading retrotransposons exhibit higher levels of CHG methylation within the retrotransposon themselves (Figure S2). Spreading levels were assessed in plants that were homozygous for mutations in the maize chromomethylase *zmet2* (GRMZM2G025592) gene, which contributes substantially to CHG methylation [48]–[49]. While there were examples of locus-specific alterations in DNA methylation levels in this mutant, there was no evidence for a reduction in the spreading of DNA methylation in low copy sequences flanking spreading retrotransposon families (Figure 2).

### Analysis of empty sites

The observation that certain families of retrotransposons have high levels of heterochromatic modifications in adjacent regions could reflect insertion site biases for these families or indicate that these families cause local spreading of heterochromatin. Examples of “empty” sites in the Mo17 haplotypes were identified and used to assess whether the high levels of DNA methylation would be observed in these regions when the retrotransposon was absent. Mo17 whole-genome shotgun WGS) sequences (generated by the DOE's Joint Genome Institute (JGI) and downloaded from ftp://ftp.jgipsf.org/pub/JGI_data/Zea_mays_Mo17/) were aligned to the B73 reference genome sequence. Empty sites were defined as being those as which at least three Mo17 sequence reads cover a low-copy sequence flanking an insertion but do not align to the retrotransposon itself and for which no Mo17 reads cover the junction between the low-copy sequence and the retrotransposon. In total, 668 empty sites were identified for the spreading (both) retrotransposon families and 29 empty sites for the spreading (H3K9) retrotransposon families for which we had DNA methylation data in the unique regions flanking the insertion. The lack of the specific insertion in Mo17 was confirmed at 13 of the 14 empty sites that were tested using site-specific PCR primers to confirm the presence/absence of specific insertions. This suggests that there is a low false-positive rate in the identification of empty sites in Mo17. However, given the low coverage of the WGS data and challenges associated with aligning polymorphic sequences it is likely that many of the true empty sites were not identified in this analysis.

**Figure 3:**
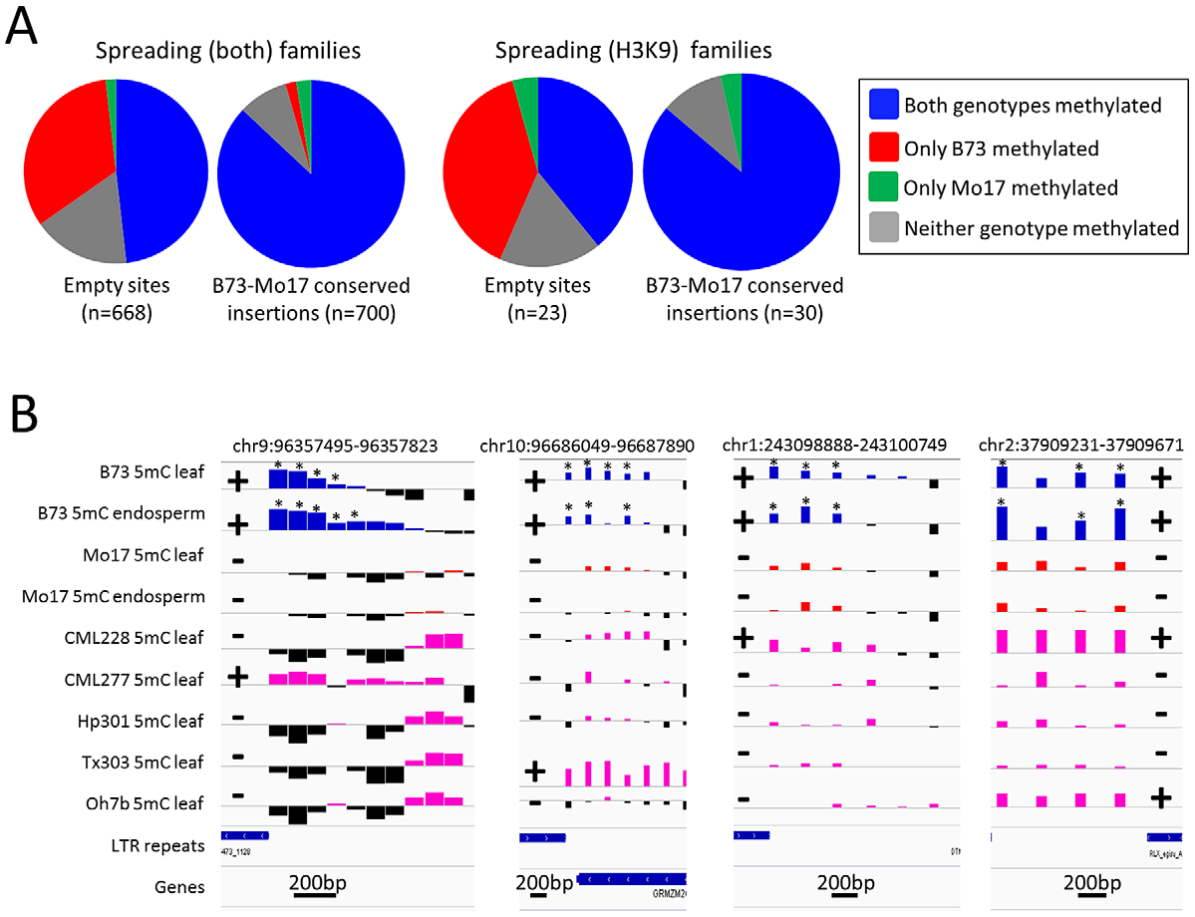
Heterochromatic marks are associated with presence of retrotransposons. (A) Whole genome shotgun sequence data for Mo17 was used to identify retrotransposon insertions that are present within the B73 haplotype but missing in Mo17. The relative level of DNA methylation at the probe nearest the empty site was assessed in B73 and Mo17 for empty sites of spreading (both) and for spreading (H3K9) retrotransposon families. For each set of families we compared the distribution of methylation patterns to a similar number of insertion sites that are conserved in both B73 and Mo17. (B) The DNA methylation levels for four of these “empty” sites (the coordinates specify the retrotransposon present in B73) are shown for two tissues of B73 (blue bars) and Mo17 (red bars) as well as for a single tissue of five other maize genotypes; CML228, CML277, Hp301, Tx303 and Oh7b (pink bars). Black bars for all genotypes indicate depletion of methylation signal. The location of the retrotransposon and its presence or absence are indicated by + or − symbols, respectively. The genotypes containing the insertion of the retrotransposon all exhibit enrichment for DNA methylation while the genotypes without the insertion do not. The “*” indicates probes with significantly (P<0.01) higher DNA methylation levels in B73 relative to the same tissue in Mo17. The horizontal gray line indicates the genome-wide average for each of the modifications. Higher values indicate higher levels of DNA methylation and all plots are show on the same scale for the y-axis. The scale for black line near the bottom of each plot indicates the base pair scale for the x-axis.

The level of DNA methylation at the probe nearest to the empty site was used to assess relative DNA methylation levels with (B73) and without (Mo17) each insertion (Figure 3). The low-copy DNA flanking many of the empty sites showed differences in DNA methylation levels between B73 and Mo17 in 34.7% of the empty sites flanking spreading (both) retrotransposons and in 43.5% of the empty sites flanking spreading (H3K9) retrotransposons (Figure 3A). Over 95% of the empty sites with differential methylation had higher DNA methylation levels in B73 (the genotype with the insertion) than in Mo17. While 35–43% of the probes flanking the empty sites for spreading retrotransposons had variable DNA methylation in B73 and Mo17, only 3% of genome-wide probes assayed show significantly different levels of DNA methylation in B73 and Mo17 and these differences include equal frequencies of higher methylation levels in each genotype. This suggests that the insertion of the retrotransposon conditioned higher levels of DNA methylation and was responsible for the observed DNA methylation polymorphisms. In contrast, DNA methylation levels were similar (and frequently quite high) between B73 and Mo17 when the retrotransposon insertion was present in both genotypes (Figure 3A). Closer inspection of several of the empty sites provides evidence for enrichment of DNA methylation or H3K9me2 in regions flanking the sites in B73 but these modifications were not observed in the Mo17 haplotype that lacks the retrotransposon (Figure 3B). The presence of the insertion as well as the enrichment for DNA methylation was also assessed in five other inbred genotypes of maize (Figure 3B). The presence of insertions was strongly correlated with the presence of high levels of DNA methylation in these other genotypes as well. These results suggest that the high level of heterochromatin observed around these spreading retrotransposon families is an outcome of TE insertion rather than insertion site bias.

### Characterization of retrotransposon families that induce local spreading of heterochromatin

**Figure 4:**
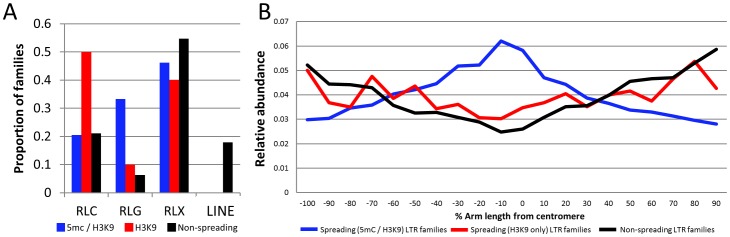
Characterization of retrotransposons that exhibit heterochromatin spreading. (A) The proportion of families within each of the superfamily designations [RLC – copia; RLG – gypsy; RLX – unknown retrotransposon; LINE – LINE elements] is shown for retrotransposons with spreading of both DNA methylation and H3K9me2 (blue), families with H3K9 spreading only (red) and families without spreading (black). (B) The relative abundance of the retrotransposons within each category was determined according to chromosomal position. The retrotransposons in families with spreading (both) are found throughout the maize chromosomes but are enriched in pericentromeric regions relative to the other families. The y-axis provides a normalized estimate of TE abundance along the chromosome (normalized relative to total copy number for each family).

The finding that only a subset of maize class I retrotransposon families are associated with local spreading of heterochromatin suggested that there might be intrinsic differences among different retrotransposon families that would explain this variation. We proceeded to characterize these families to ascertain whether there were specific common attributes of spreading families. None of the LINE families exhibit evidence for spreading of heterochromatic marks. RLG (gypsy) families are over-represented among spreading (both) retrotransposon families, while the spreading (H3K9) retrotransposons have more RLC (copia) families than expected (Figure 4A). Spreading (both or H3K9) retrotransposons exhibit significantly higher copy number and comprise a greater fraction of the genome (Table S1, attributes from [29]) than do non-spreading retrotransposon families (Figure S10A–S10B). While there are significant differences in copy number and total Mb within the genome there are examples of families with spreading that have lower copy numbers (Figure S10A). In addition, spreading (both) retrotransposon families have significantly higher average fragment lengths than do non-spreading families (Table S1). Spreading families do not have a significant difference in their mean insertion date relative to non-spreading families (Table S1). However, the analysis of average insertion date for each family (Figure S10C) shows that while non-spreading retrotransposon families include both old and young families the spreading (both) retrotransposon families only include younger families. The analysis of several characteristics of the retrotransposon families with and without spreading provides evidence for some significant differences but none of these factors are sufficient for predicting whether or not spreading occurs. Previous studies that had assessed expression of some retrotransposons in maize tissues [50]–[51] did not find unusually high or low abundance for transcripts of the families with heterochromatin spreading relative to other families.

The relative abundance of spreading (both) retrotransposons is higher in the middle of the chromosome than the other families suggesting that these retrotransposons may be enriched in pericentromeric regions (Figure 4B). However, it should be noted that there are other retrotransposon families also preferentially located in pericentromeric regions [29] but that do not show spreading of heterochromatin to low-copy adjacent regions. Hence, the pericentromeric enrichment is insufficient for heterochromatin spreading. The observation that the spreading (both) retrotransposon families are enriched in pericentromeric regions suggested the possibility that the higher levels of DNA methylation in flanking sequences may be due to sampling bias. Because pericentromeric regions tend to have higher levels of DNA methylation [39] it is possible that higher sampling of these regions led to the observation of spreading. However, an analysis of the levels of DNA methylation in low-copy flanking regions relative to chromosome position provides evidence that low-copy sequences flanking spreading (both) retrotransposons is substantially higher than the corresponding regions flanking non-spreading families throughout the chromosome in both CG and CHG contexts (Figure S11). The levels of CG and CHG DNA methylation in spreading (H3K9) retrotransposon families are intermediate (Figure S11).

### Genes located near retrotransposon with spreading of heterochromatic marks tend to have lower expression

**Figure 5:**
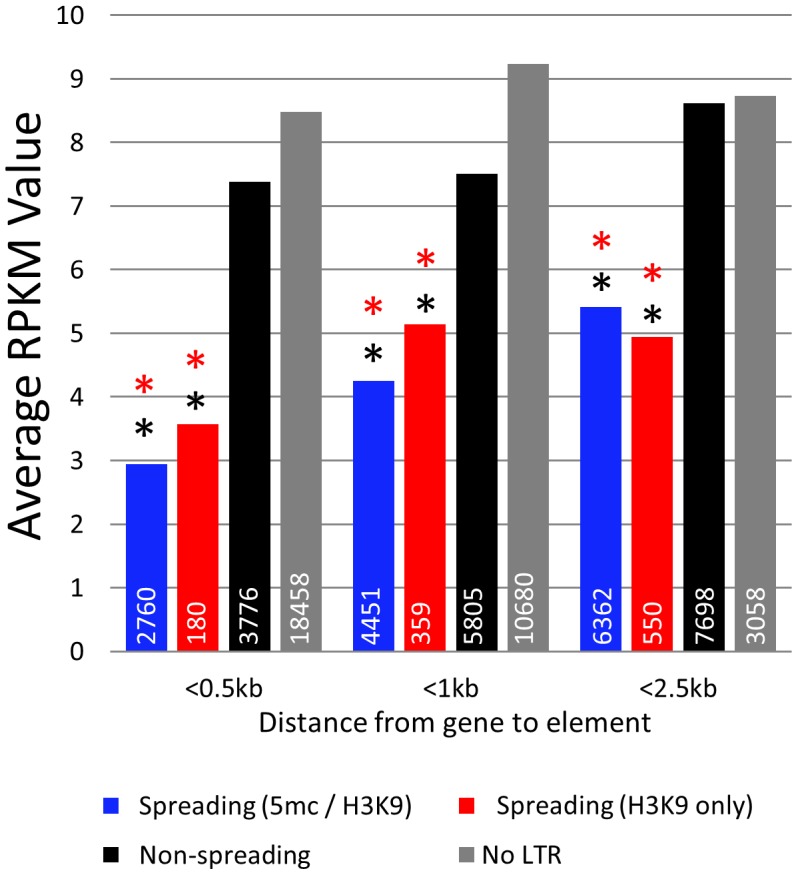
Genes near spreading retrotransposons show lower expression than genes near non-spreading retrotransposons. Average RPKM values (from B73 leaf) for all genes falling within 0.5 kb, 1 kb, and 2.5 kb from the respective class of retrotransposons were developed. White numbers within bars indicate the total number of genes within each category. Red asterisks indicate highly significant (p<0.001) difference from the non-spreading retrotransposons (black) within the distance classification. Black asterisks indicate significant (p<0.001) difference from genes not near any retrotransposons (grey).

The finding that some retrotransposon families exhibit spreading of heterochromatic marks to surrounding sequences while others do not led us to hypothesize that these families may influence expression of nearby genes. RNAseq was used to estimate transcript abundance in three tissues of B73 and Mo17 including the identical leaf tissue samples used for profiling DNA methylation levels. All maize genes were annotated to identify the first retrotransposon 5′ of the transcription start site and to determine the distance between the retrotransposon and the transcription start site. Genes that are located near retrotransposons that exhibit spreading (both or H3K9) have significantly (p<0.001) lower expression levels in all genotypes and tissue examined (Figure 5; Figure S12A). This reduction in expression is most severe when we examine genes with retrotransposons inserted within 500 bp of the transcription start site. As the distance between the insertion site and the transcription start site increases there is less evidence for an effect on expression levels, suggesting a limited range within which retrotransposons can influence gene expression. The genes located near spreading (both) and spreading (H3K9) retrotransposons frequently have no detectable expression (Figure S12B). However, even if we exclude genes with no expression, the mean expression of genes near spreading retrotransposons is lower (p<0.001) (Figure S12C).

## Discussion

Epigenetic variation in low-copy sequences can be the result of pure epigenetic changes (no correlation with DNA sequence polymorphisms) or occur in a facilitated or obligatory fashion such that DNA sequence differences contribute to the epigenetic changes [10]. A handful of examples in which epigenetic differences that impact phenotype has been shown to involve TEs inserted near genes [13]–[19], [52] and genomic profiling of DNA methylation in Arabidopsis has revealed some examples of heterochromatin spreading from TEs [20], [21]. However, it has not been clear whether all TEs have similar effects on neighboring chromatin or whether there are family-specific attributes that affect the spreading of heterochromatin. A recent study analyzed several families of retrotransposons in mouse and found that there is variation in the level of heterochromatin spreading [24] and there have been suggestions of variation in the effects of different repetitive elements on nearby gene expression in Arabidopsis [22], [23]. The complex organization of the maize genome with interspersed TEs and genes provides the opportunity to examine differences among class I retrotransposon families. The chromatin state of any low-copy region of a genome is likely influenced by nearby sequences including regulatory elements and insulator elements. In addition, it is quite likely that TEs will exert an influence on the chromatin state. By examining the average level of chromatin modifications in low-copy sequences neighboring families of retrotransposons we found evidence for heterochromatic spreading from a subset of the moderate to high-copy retrotransposon families in maize. Even in these families the heterochromatic marks spread only 600–1,000 base pairs from the retrotransposon. It is worth noting that there may be other mechanisms through which retrotransposons influence flanking regions. Our assessment is based upon only two chromatin marks, H3K9me2 and DNA methylation. These marks are frequently associated with heterochromatin, but there may be other specific types of chromatin marks that spread from these and transposon families.

There is also evidence that differences in interspecific variation in transposon insertions contributes to gene expression diversity between related species [22], [23]. Here we provide evidence that transposon insertions can also contribute to differences in DNA methylation patterns and gene expression levels within a species. Many TE insertions are exhibit presence/absence variation among maize haplotypes [31]–[34]. The retrotransposons that cause spreading of heterochromatin are expected to result in obligatory epigenetic variation in the low-copy sequences that flank insertions. Indeed, we found that the levels of DNA methylation and H3K9me2 were quite different in B73 and Mo17 at regions that exhibit presence/absence variation for an insertion of a retrotransposon from one of the spreading families. Specifically, these retrotransposons with spreading of heterochromatin may contribute to obligatory and facilitated epialleles, as defined by Richards [10], among different genotypes. Genomic resequencing is often used to identify SNPs as a means to explain phenotypic variation. However, it might be important to also use resequencing data to identify retrotransposon insertion polymorphisms, especially for the retrotransposon families that exhibit spreading of heterochromatic marks. The polymorphism for these insertions may lead to functional variation in the expression of nearby genes.

Barbara McClintock proposed the concept that transposons could serve as “controlling” elements that would influence nearby genes [6]–[7] and this could be extended to include the potential for retrotransposons to influence nearby genes as well. There are examples in which transposons contain regulatory elements or cryptic promoters that can influence the expression of nearby genes [9], [53]. There is also evidence that some transposons can act as controlling elements by “seeding” heterochromatin that spreads to adjacent low copy sequences [10]–[12]. Here we have shown that this activity is not a generic feature of all retrotransposons but is instead limited to a subset of retrotransposons. Hollister and Gaut [22] provide evidence that the presence of heavily silenced TEs near genes may lead to reduced expression and result in fitness consequences. This would suggest that many TEs would evolve to have minimal effects on neighboring genes to reduce their fitness costs. There is evidence that some *Drosophila* retrotransposons contain insulator elements that reduce the spreading of chromatin states [54]. Alternatively, studies at the *bns* locus in Arabidopsis have suggested the presence of an active mechanism to prevent the spreading of heterochromatin from retrotransposons [55]. It might be expected that different families of TEs would vary in their ability to limit potential spreading of heterochromatin through the presence of insulators or the recruitment of factors that limit spreading. Hollister and Gaut [22] noted heterogeneity among families of Arabidopsis class I retrotransposons for their distance to the nearest gene and suggested that this may reflect family specific differences in heterochromatin spreading. The analysis of the large families of retrotransposons in maize permitted us to identify several families of retrotransposons with high levels of spreading. These retrotransposon families may be considered as bad “neighbors” for genes. Indeed we find that many genes located near retrotransposons with spreading tend to be silenced or expressed at lower levels. We might predict that insertions of retrotransposons from these families will be more strongly selected against when inserted near genes, especially if they affect gene expression. Therefore, our observed expression differences will only report effects that have been tolerated during natural and artificial selection of maize lines. Consistent with this possibility, our observation that these retrotransposon families are enriched in relatively gene-poor pericentromeric regions may reflect selection against insertions of these retrotransposons when they are near genes. Further research efforts to understand the basis of this difference will be important in providing the ability to predict which retrotransposon families are likely to condition spreading of heterochromatin and understanding the consequences of the spreading of heterochromatin.

## Materials and Methods

### Epigenomic profiling

DNA methylation profiling on three replicates of 3^rd^ leaf tissue of B73 and Mo17 was performed as described [39 – GSE29099]. Briefly, methylated DNA was immunoprecipitated with an anti-5-methylcytosine monoclonal antibody from 400 ng sonicated DNA using the Methylated DNA IP Kit (Zymo Research, Orange, CA; Cat # D5101). For each replication and genotype, whole genome amplification was conducted on 50–100 ng IP DNA and also 50–100 ng of sonicated DNA (input control) using the Whole Genome Amplification kit (Sigma Aldrich, St. Louis, MO, Cat # WGA2-50RXN). For each amplified IP input sample, 3 ug amplified DNA were labeled using the Dual-Color Labeling Kit (Roche NimbleGen, Cat # 05223547001) according to the array manufacturer's protocol (Roche NimbleGen Methylation UserGuide v7.0). Each IP sample was labeled with Cy5 and each input/control sonicated DNA was labeled with Cy3. H3K9me2 and H3K27me3 profiling were performed on three replicates of B73 and Mo17 seedlings using antibodies specific for H3K27me3 (#07-449) and H3K9me2 (#07-441) purchased from Millipore (Billerica, USA). For each replicate, 1 g of plant material was harvested on ice, rinsed with water, and crosslinked with 1% formaldehyde for 10 minutes under vacuum. Cross-linking was quenched by adding glycine solution to a final concentration of 0.125 M under vacuum infiltration for 5 minutes. Treated tissue was frozen in liquid nitrogen and stored at −800 C until chromatin extraction. Chromatin extractions were performed using EpiQuik Plant ChIP Kit (Epigentek, Brooklyn, USA) according to manufacturer's recommendations. Extracted chromatin was sheared in 600 µl of the EpiQuik buffer CP3F with 5 10-second pulses on a sonicator. To test and optimize sonication conditions, cross-linking was reversed in a sample of sheared chromatin and the resulting products were analyzed on agarose gel. Sonication conditions were optimized to yield predominantly 200–500 bp DNA samples. Chromatin immunoprecipitations, reverse cross-linking, and DNA cleanup was performed using EpiQuik Plant ChIP Kit (Epigentek) according to manufacturer's recommendations. For each genotype, antibody, and replicate, 50–100 ng of input and immunoprecipitated (IP) DNA was amplified with a whole genome amplification kit (WGA2, Sigma, St. Louis, USA). The amplification of no antibody control (negative control) was always 5–10 fold less efficient confirming specificity of immunoprecipitation. For each amplified IP and input sample, 3 ug of amplified DNA were labeled using the Dual-Color Labeling Kit (Roche NimbleGen, Cat # 05223547001) according to the array manufacturer's protocol (Roche NimbleGen Methylation User- Guide v7.0). Each IP sample was labeled with Cy5 and each input/control sonicated DNA was labeled with Cy3. Samples were hybridized to the custom 2.1 M probe array (GEO Platform GPL13499) for 16–20 hrs at 42 C. Slides were washed and scanned according to NimbleGen's protocol. Images were aligned and quantified using NimbleScan software (Roche NimbleGen) producing raw data reports for each probe on the array. The histone modification and methylation mutants array data can be obtained from GEO accession (GSE39460). The resulting microarray data were imported into the Bioconductor statistical environment (http://bioconductor.org/). Microarray data channels were assigned the following factors: B73 immunoprecipitation, Mo17 immunoprecipitation, B73 input, or Mo17 input depending on sample derivation. Non-maize probes and vendor-supplied process control probes were configured to have analytical weights of zero. Variance-stabilizing normalization was used to account for array-specific effects. Factor-specific hybridization coefficients were estimated by fitting fixed linear model accounting for dye and sample effects to the data using the limma package [56]. The probes were each annotated with respect to their location relative to repeats from the ZmB73_5a_MTEC_repeats file available from www.maizesequence.org. Each probe was only associated with the closest repeat and all probes located within 5 kb of a repeat were retained for further analyses. The probes were assigned based on distance to the retrotransposon and include both upstream (5′) and downstream (3′) sequences together. The distribution of retrotransposons along the length of the chromosome was performed as described in [57]. Data formatted for the Integrative Genomics Viewer (IGV) can be downloaded from http://genomics.tacc.utexas.edu/data/rte_methylation_spreading/.

### Bisulphite sequencing

DNA was extracted from the outer tissues of B73 ears whose silks had emerged but had not been fertilized. Sodium bisulfite-treated Illumina sequencing libraries were prepared using a method similar to that of Lister et al [58]. Alignment to the genome (AGPv2) and identification of methylated cytosines was performed using BS Seeker [59]. A total of 198,333,982 single-end reads with unique alignments specifically on the ten chromosomes were obtained, with an average genome-matching read length of 72.8 bases (7.0× coverage, SRA accession SRA050144.1). The level of methylation in CG, CHG and CHH contexts and the total proportion of DNA methylation was calculated for non-repeat masked sequences (as annotated within ZmB73_5a_MTEC_repeats) located within 1 kb of each retrotransposon family. Percent methylation is defined as the number of methylated Cs per total number of Cs for a region. BEDTools [60] was used to identify low-copy sequences flanking retrotransposons.

### Identification and analysis of empty sites

Approximately 63M Mo17 454 whole-genome shotgun sequencing reads generated by the DOE's Joint Genome Institute (JGI) were trimmed and aligned to Maize B73 reference genome (AGPv2) and reads aligned uniquely (single loci) were filtered for subsequent analysis. A retrotransposon insertion site was classified as empty if we identified at least 3 WGS reads supporting the site that aligned to the insertion site that included>50 bp of aligned sequence outside of the repeat region in B73 with similarity of ≥94%, relatively short unaligned tails (≤20 bp), and contained a long overhang of >20 bp that begins ±3 bp from the annotated retrotransposon insertion site. PCR primers were designed to amplify the sequence at the “empty” sites using the B73 sequence (which contained the insertion) and the Mo17 sequence (which lacks the insertion) (Table S2). These same primers were also used to assess the presence or absence of the insertion in several other maize genotypes including CML228, CML277, Hp301, Tx303 and Oh7b. Seeds for these genotypes were obtained from the USDA North Central Regional Plant Introduction Station. PCR and gel electrophoresis was conducted as described [61].

### RNA–seq and expression analysis

RNA–seq was performed on three biological replicates of four tissues (3^rd^ leaf, embryo, endosperm, and immature ear) for both B73 and Mo17. Samples were prepared at the University of Minnesota BioMedical Genomics Center in accordance with the TruSeq library creation protocol (Illumina). Samples were sequenced on the HiSeq 2000 developing 6–17 million reads per replicate. Raw reads were filtered to eliminate poor quality reads using CASAVA (Illumina). Transcript abundance was calculated by mapping reads to the maize reference genome (AGPv2) using TopHat under standard parameters [62]. Counts of mapped reads across the exon space of the maize genome reference working gene set (ZmB73_5a) were developed using ‘BAM to Counts’ within the iPlant Discovery Environment (www.iplantcollaborative.org). RPKM values were calculated per gene. All genes within 500, 1000, 2500, and 5000 bases of the closest upstream annotated transposable element (ZmB73_5a) using BEDtools [60] were grouped by the spreading class of the nearest TE: spreading (5mc/H3K9), spreading (H3K9 only), non-spreading, and no TE within distance. Genes were also classified as expressed for any RPKM value >0. The proportions of genes showing expression for each distance and spreading class combination were calculated. Average RPKM values for each distance and spreading class combination were also calculated. Significance testing was performed non-parametrically through Wilcox rank-sum tests. Sequencing data is available from the NCBI short read archive under studies SRP013432 and SRP009313.

## Supporting Information

Figure S1 Validation of H3K9me2 ChIP-chip. (A) The efficient enrichment of DNA associated with H3K9me2 was assessed using qPCR. The copia sequence is known to be enriched for H3K9me2 while the GAPC sequence is not associated with H3K9me2 (Haring et al., 2007). Primers for these regions were used to perform qPCR using three technical replicates. The percent of input DNA recovered after IP with the H3K9me2 antibody or a noIG control was determined for both sequences. (B) Several regions were selected for validation based on ChIP-chip profiling. Two regions enriched for H3K9me2 (H1 and H2) and four regions with no evidence for H3K9me2 (L1, L2, L3, L5) were used to design primers for qPCR. The percent of input DNA recovered by ChIP using an H3K9me2 antibody or a noIG control was determined for three replicates of B73 using these primers. The H1 and H2 sequences were enriched by ChIP while the L1, L2, L3 and L5 sequences showed much lower levels of recovery.
(TIF)

Figure S2 Levels of DNA methylation within retrotransposons. Whole-genome bisulphite sequencing data was used to assess the average level of methylation within retrotransposons. DNA methylation levels within each sequence context (CG, CHG and CHH) were determined for each family of retrotransposon. The average levels of methylation for elements classified as having spreading of both 5 mC and H3K9me2, spreading of H3K9me2 only and non-spreading were determined and plotted. The error bars indicate standard deviation among retrotransposon families and “*” indicate significant (p<0.001) differences for a group relative to the non-spreading families. The level of internal methylation at CG sites is similar for retrotransposons with and without spreading of heterochromatin although there is a significant difference in spreading (both) relative to non-spreading families. CHG methylation is slightly lower in non-spreading families. The non-spreading families have slightly elevated levels of CHH methylation relative to the other families.
(TIF)

Figure S3 Chromatin modifications in regions flanking maize retrotransposon superfamilies including RIX – LINE (black), RLC – copia (red), RLX – unknown LTR (blue) and RLG – gypsy (green). We identified probes located in low-copy DNA flanking retrotransposons in maize. The number of probes for each class is indicated within the Figure legend. The average level of DNA methylation (A–B), H3K9me2 (C) or H3K27me3 (D) is shown for the 5,000 bp adjacent to each superfamily. The level of chromatin modifications are based on ChIP-chip experiments and the y-axis represents the average log ratio for the immunoprecipitated samples relative to genomic input DNA.
(TIF)

Figure S4 Profiles of chromatin surrounding spreading (both) retrotransposons. Black lines indicate DNA methylation. Blue lines indicate H3K9. Green lines indicate H3K27 levels. Chromatin values calculated include probes flanking both ends of retrotransposable elements. Copy number of repeat fragments in the B73 reference genome as well as the total number of probes flanking each repeat are displayed. The y-axis provides the distance (in bp) from the retrotransposon insertion.
(TIF)

Figure S5 Profiles of chromatin surrounding spreading (H3K9) retrotransposons. Black lines indicate DNA methylation. Blue lines indicate H3K9. Green lines indicate H3K27 levels. Chromatin values calculated include probes flanking both ends of retrotransposable elements. Copy number of repeat fragments in the B73 reference genome as well as the total number of probes flanking each repeat are displayed. The y-axis provides the distance (in bp) from the retrotransposon insertion.
(TIF)

Figure S6 Similar profiles of DNA methylation enrichment adjacent to retrotransposon families in different tissues and genotypes of maize. The relative level of DNA methylation in each 200 bp bin is shown for each of the 144 retrotransposon families. The red color indicates enrichment for the modification while blue indicates depletion of the mark. Black indicates levels of the modification similar to genome-wide average values. The color intensity is based on the average log ratio of immunoprecipitated DNA relative to input DNA. The retrotransposons are grouped according to whether they show spreading for DNA methylation and H3K9me2, just H3K9me2 or neither of the marks. The profiles are shown for B73 leaf (A), Mo17 leaf (B), B73 endosperm (C) and B73 embryo (D).
(TIF)

Figure S7 Histone modification patterns in different genotypes. The profile of several histone modifications is shown for both B73 and Mo17. The relative level of H3K9(di)- or H3K27(tri)-methylation in each 200 bp bin is shown for each of the 144 retrotransposon families. Modification levels calculated include probes flanking both ends of retrotransposable elements. The red color indicates enrichment for the modification while blue indicates depletion of the mark. Black indicates levels of the modification similar to genome-wide average values. The color intensity is based on the average log ratio of immunoprecipitated DNA relative to input DNA. The retrotransposons are grouped according to whether they show spreading for DNA methylation and H3K9me2, just H3K9me2 or neither of the marks. (A) and (B) display the profiles for H3K9me2 in B73 and Mo17 seedlings, respectively. (C) and (D) display the profiles for H3K27me3 in B73 and Mo17 seedlings, respectively. (E) shows the H3K27me3 profile for immature ear tissue from B73.
(TIF)

Figure S8 Similar levels of H3K9me2 within spreading and non-spreading retrotransposons. The ChIP-chip assay does not provide information on the abundance of H3K9me2 within repetitive regions. In order to assess whether the level of H3K9me2 was similar within retrotransposons with, and without, spreading we designed primers for internal sequences of 10 retrotransposon families including six with spreading (both) and four that were classified as non-spreading. The qPCR protocol described by Haring et al. (2007) was used to assess the percent input DNA recovered by immunoprecipitation. The percent of input DNA recovered after IP with the H3K9me2 antibody or a noIG control was determined for both sequences and the standard deviation is indicated with error bars. There were high levels of H3K9me2 in each of these retrotransposons but there was no significant difference between the spreading and non-spreading retrotransposons.
(TIF)

Figure S9 Small RNA coverage of retrotransposon families. B73 shoot small RNAs [47]. Small RNA reads for all size classes were mapped to the maize reference genome (AGPv2) using BLAT [63] under standard parameters. Coverage of small RNA reads over annotated maize transposons (ZmB73_5a) were calculated using BEDtools coverageBED [60]. (A) The average small RNA count per retrotransposon family was determined for each of the 144 retrotransposon families. The average count (and standard deviation) for families classified as spreading (both), spreading (H3K9) and non-spreading was then determined. There is no evidence for significant differences in the average small RNA count. (B) The proportion of each retrotransposon covered by small RNAs was then determined in a similar fashion. There is no significant difference in the proportion of coverage for the spreading and non-spreading families of retrotransposons. Small RNA data were downloaded from GEO as samples GSM918108 [47].
(TIF)

Figure S10 Characteristics of retrotransposon families with spreading of heterochromatic marks. In each of the plots the retrotransposon families are grouped into both (5 mC and H3K9), H3K9 only and non-spreading columns and the superfamily is indicated by the color. The data points are jittered to allow visualization of all families. (A) The genomic copy number of each family is shown using a log-scale. The families with spreading of both marks tend to have higher copy numbers. However, there is an overlap in the range of copy number for families with and without spreading. (B) The total Mb of the B73 genome comprised by each family is shown. (C) The average insertion date for each family is plotted. While the non-spreading families include both young and old retrotransposons the families with spreading of both marks tend to be younger families.
(TIF)

Figure S11 DNA methylation levels in flanking sequences are similar throughout the chromosome. The level of DNA methylation in sequences flanking retrotransposons was determined from bisulphite sequencing data. Only flanking sequences that did not contain any repetitive sequences within 1 kb of the retrotransposon were used. The proportion of methylated cytosines in CG (A) or CHG (B) contexts was determined for 1 kb of low copy sequences flanking spreading (both), spreading (H3K9) and non-spreading retrotransposon families.
(TIF)

Figure S12 Genes near spreading retrotransposons show lower expression than genes near non-spreading retrotransposons. (A) Average RPKM values (from B73 Ear, B73 Tassel, Mo17 leaf, Mo17 Ear, and Mo17 Tassel tissues) for all genes falling within 0.5 kb, 1 kb, and 2.5 kb from the respective class of retrotransposons were determined. (B) Proportion of genes expressed (RPKM>0) in B73 leaf tissue for genes near 5 mc and H3K9 spreading elements, H3k9 spreading only, non-spreading, and no TE nearby. Red asterisks indicate highly significant (p<0.001) difference from the non-spreading elements within the distance classification. Black asterisks indicate significant (p<0.001) difference from genes not near any TE. (C) Average RPKM values in B73 leaf tissue for expressed genes (all genes with RPKM = 0 were omitted) falling within 0.5 kb, 1 kb, and 2.5 kb from the respective class of retrotransposons were developed. Red asterisks indicate significant (p<0.05) difference from the non-spreading elements (green) within the distance classification. Black asterisks indicate significant (p<0.05) difference from genes not near any retrotransposons (purple).
(TIF)

Table S1 Attributes and classification of retrotransposon families. A list of 145 retrotransposon families with information and statistics about each family in the B73 genotype.
(XLSX)

Table S2 Primers used for empty site validation.
(XLSX)
