# Supplementary figures and images for: Spreading of Heterochromatin Is Limited to Specific Families of Maize Retrotransposons
Source: PLoS Genet. 2012 Dec 13;8(12):e1003127. doi: 10.1371/journal.pgen.1003127 (PMC3521669; doi:10.1371/journal.pgen.1003127)

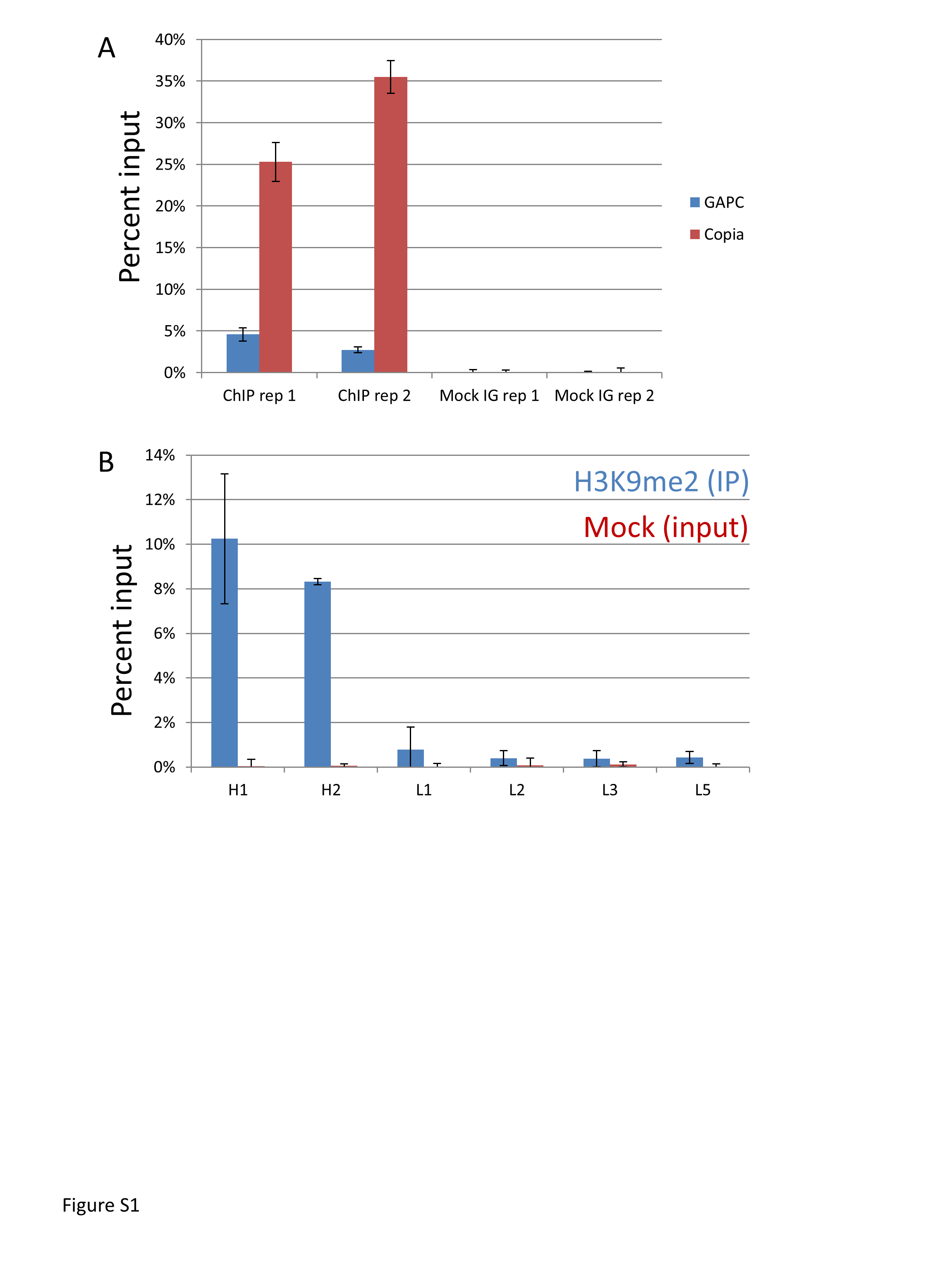

Supplement: Figure S1 — Validation of H3K9me2 ChIP-chip. (A) The efficient enrichment of DNA associated with H3K9me2 was assessed using qPCR. The copia sequence is known to be enriched for H3K9me2 while the GAPC sequence is not associated with H3K9me2 (Haring et al., 2007). Primers for these regions were used to perform qPCR using three technical replicates. The percent of input DNA recovered after IP with the H3K9me2 antibody or a noIG control was determined for both sequences. (B) Several regions were selected for validation based on ChIP-chip profiling. Two regions enriched for H3K9me2 (H1 and H2) and four regions with no evidence for H3K9me2 (L1, L2, L3, L5) were used to design primers for qPCR. The percent of input DNA recovered by ChIP using an H3K9me2 antibody or a noIG control was determined for three replicates of B73 using these primers. The H1 and H2 sequences were enriched by ChIP while the L1, L2, L3 and L5 sequences showed much lower levels of recovery. (TIF) [file pgen.1003127.s001.tif]

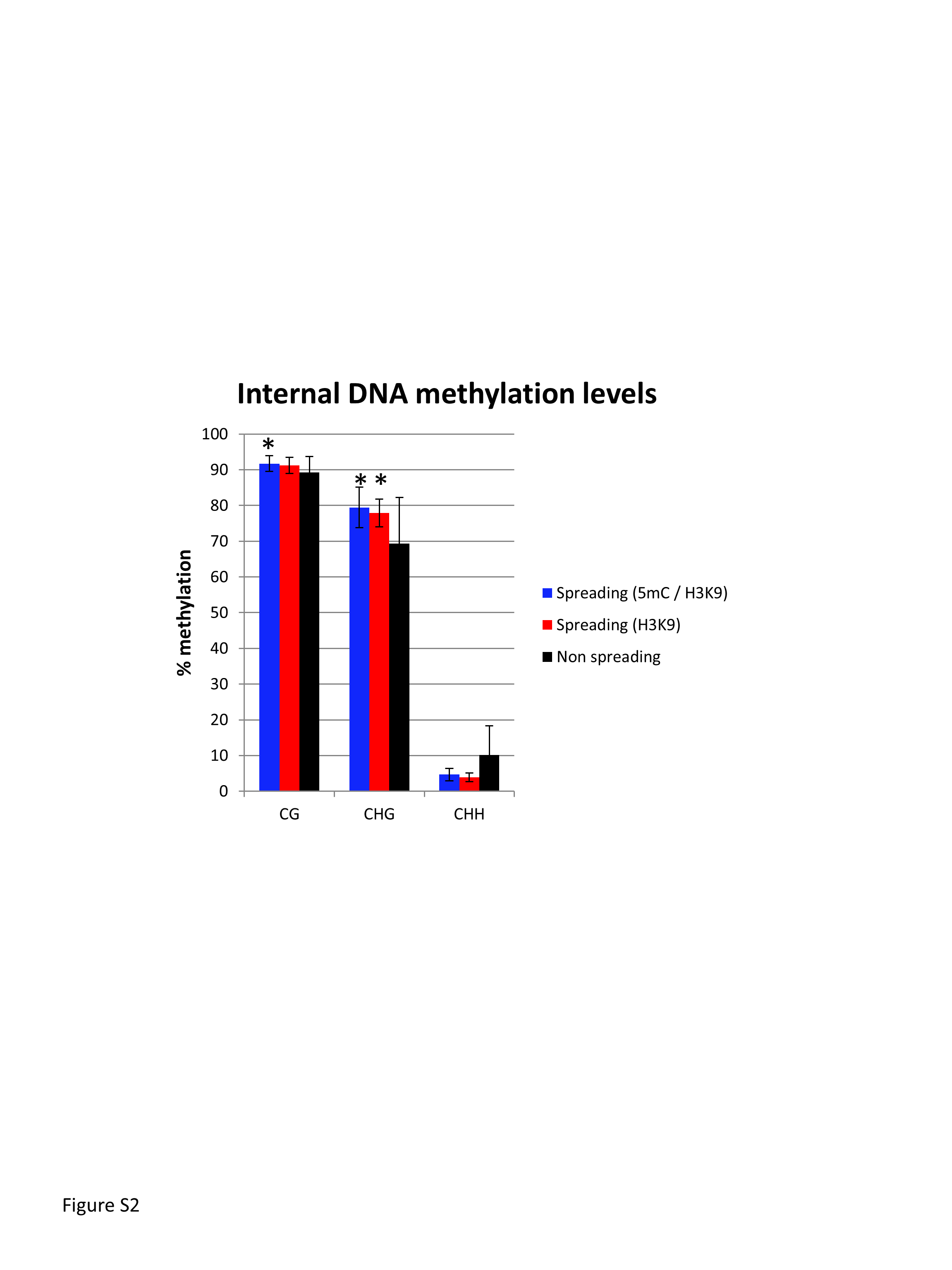

Supplement: Figure S2 — Levels of DNA methylation within retrotransposons. Whole-genome bisulphite sequencing data was used to assess the average level of methylation within retrotransposons. DNA methylation levels within each sequence context (CG, CHG and CHH) were determined for each family of retrotransposon. The average levels of methylation for elements classified as having spreading of both 5 mC and H3K9me2, spreading of H3K9me2 only and non-spreading were determined and plotted. The error bars indicate standard deviation among retrotransposon families and “*” indicate significant (p<0.001) differences for a group relative to the non-spreading families. The level of internal methylation at CG sites is similar for retrotransposons with and without spreading of heterochromatin although there is a significant difference in spreading (both) relative to non-spreading families. CHG methylation is slightly lower in non-spreading families. The non-spreading families have slightly elevated levels of CHH methylation relative to the other families. (TIF) [file pgen.1003127.s002.tif]

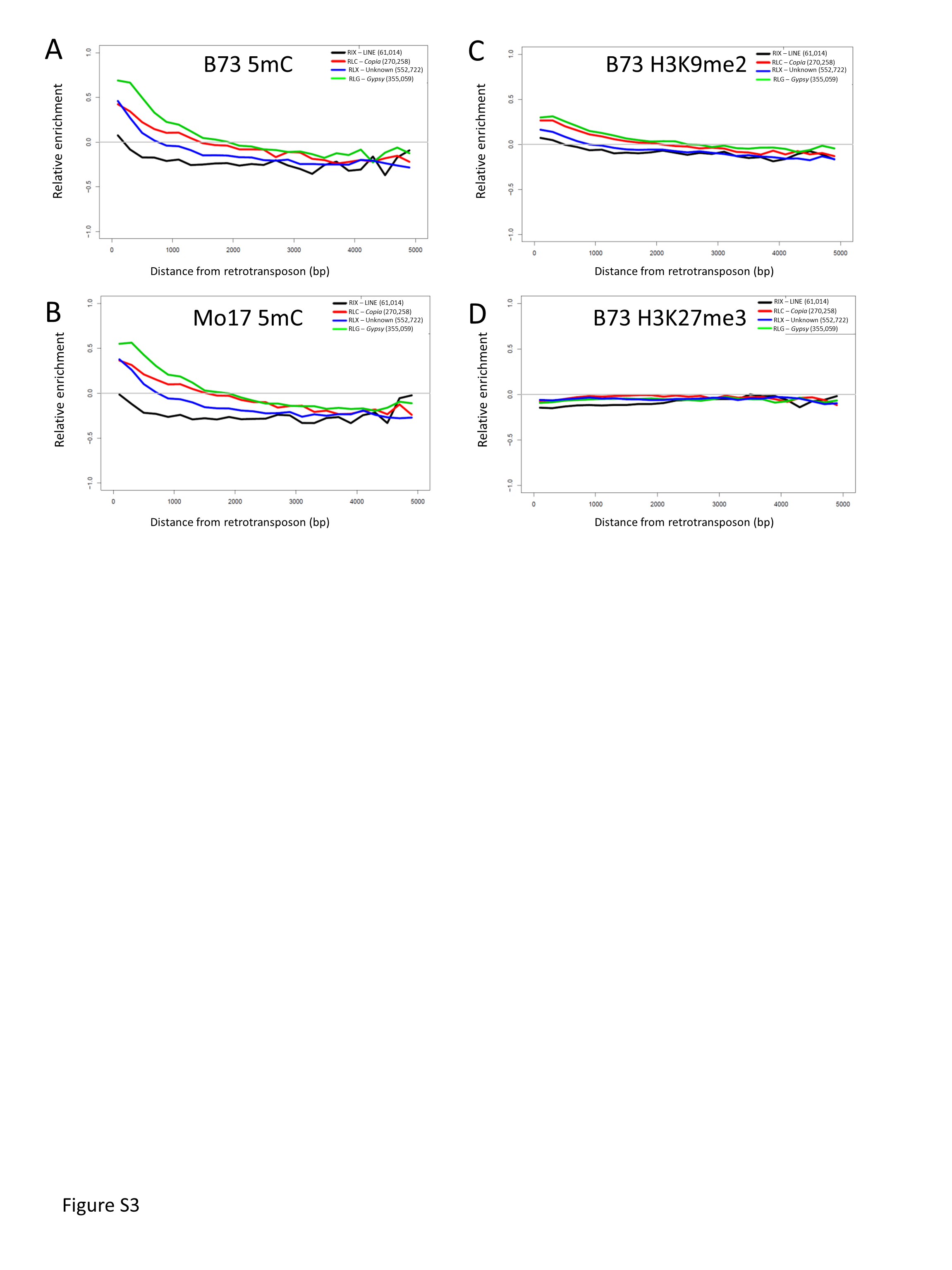

Supplement: Figure S3 — Chromatin modifications in regions flanking maize retrotransposon superfamilies including RIX – LINE (black), RLC – copia (red), RLX – unknown LTR (blue) and RLG – gypsy (green). We identified probes located in low-copy DNA flanking retrotransposons in maize. The number of probes for each class is indicated within the Figure legend. The average level of DNA methylation (A–B), H3K9me2 (C) or H3K27me3 (D) is shown for the 5,000 bp adjacent to each superfamily. The level of chromatin modifications are based on ChIP-chip experiments and the y-axis represents the average log ratio for the immunoprecipitated samples relative to genomic input DNA. (TIF) [file pgen.1003127.s003.tif]

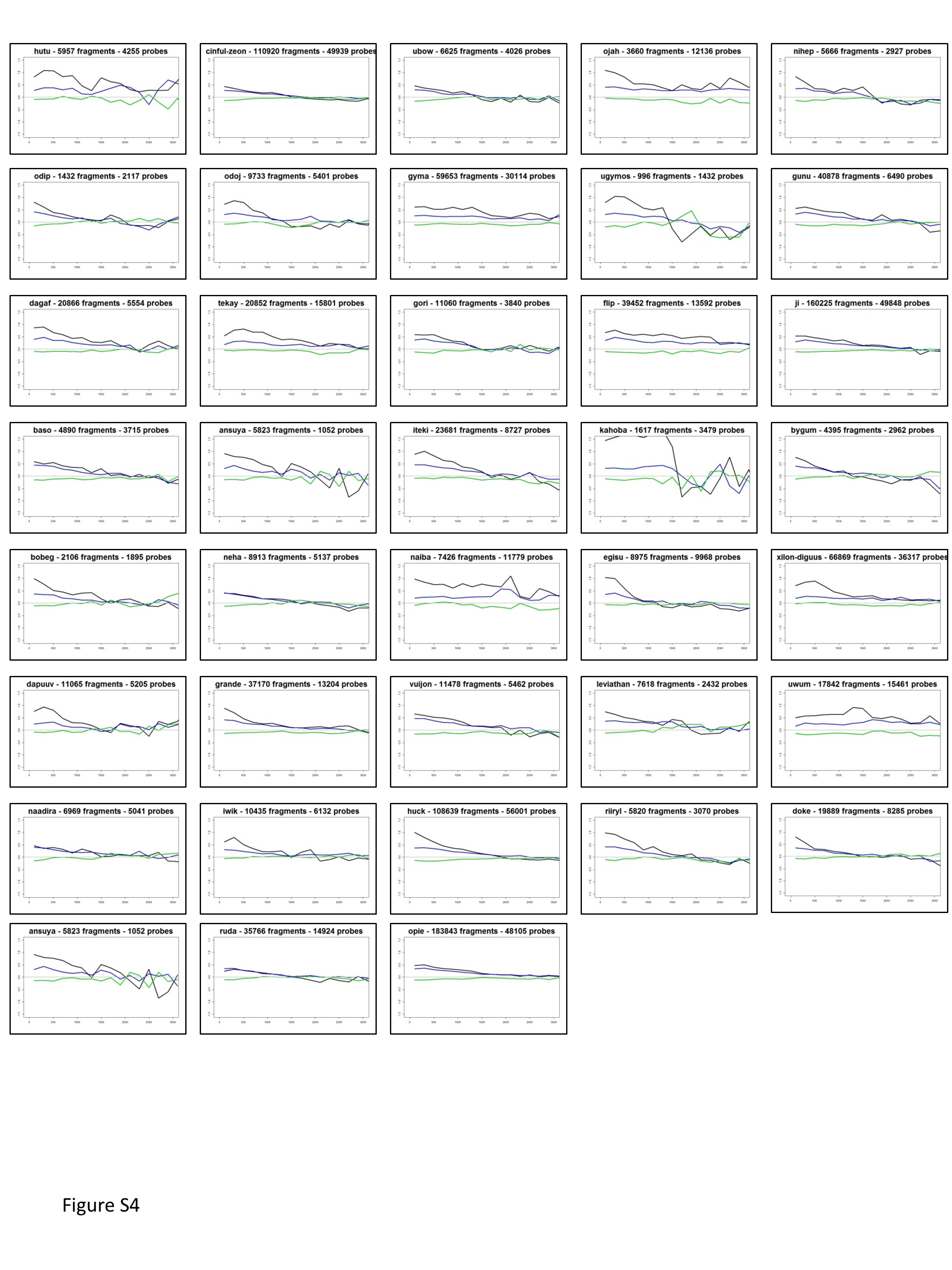

Supplement: Figure S4 — Profiles of chromatin surrounding spreading (both) retrotransposons. Black lines indicate DNA methylation. Blue lines indicate H3K9. Green lines indicate H3K27 levels. Chromatin values calculated include probes flanking both ends of retrotransposable elements. Copy number of repeat fragments in the B73 reference genome as well as the total number of probes flanking each repeat are displayed. The y-axis provides the distance (in bp) from the retrotransposon insertion. (TIF) [file pgen.1003127.s004.tif]

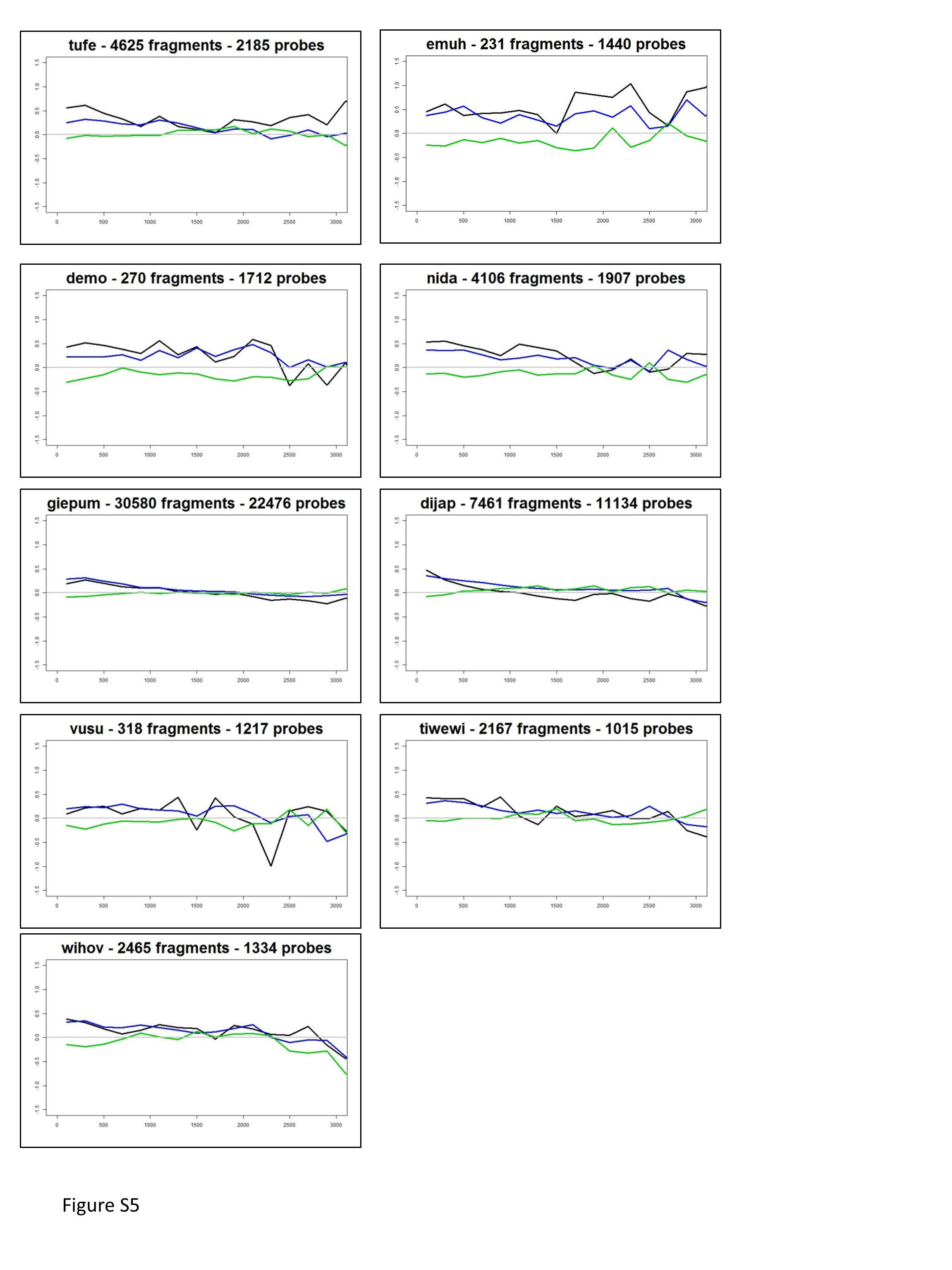

Supplement: Figure S5 — Profiles of chromatin surrounding spreading (H3K9) retrotransposons. Black lines indicate DNA methylation. Blue lines indicate H3K9. Green lines indicate H3K27 levels. Chromatin values calculated include probes flanking both ends of retrotransposable elements. Copy number of repeat fragments in the B73 reference genome as well as the total number of probes flanking each repeat are displayed. The y-axis provides the distance (in bp) from the retrotransposon insertion. (TIF) [file pgen.1003127.s005.tif]

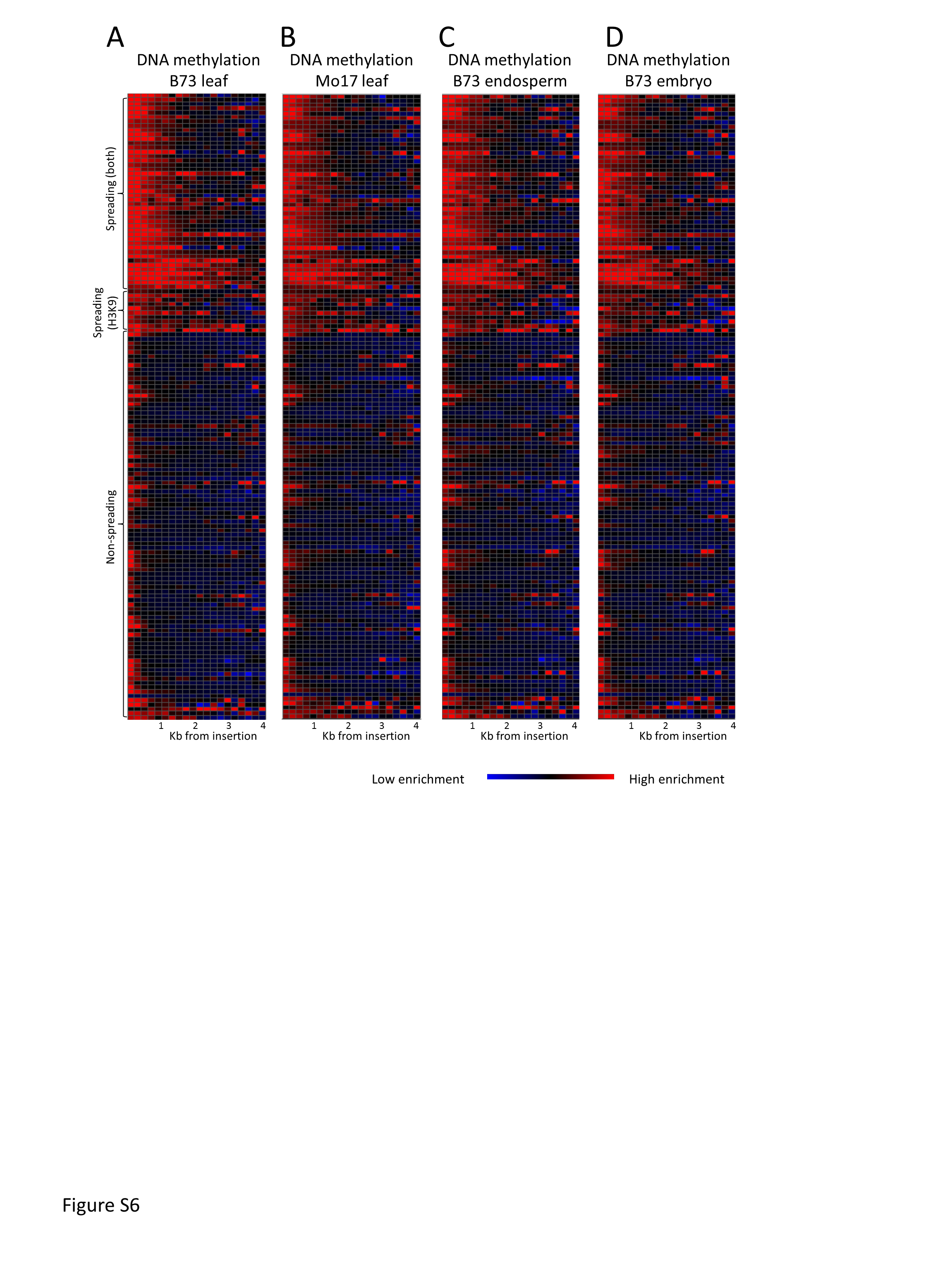

Supplement: Figure S6 — Similar profiles of DNA methylation enrichment adjacent to retrotransposon families in different tissues and genotypes of maize. The relative level of DNA methylation in each 200 bp bin is shown for each of the 144 retrotransposon families. The red color indicates enrichment for the modification while blue indicates depletion of the mark. Black indicates levels of the modification similar to genome-wide average values. The color intensity is based on the average log ratio of immunoprecipitated DNA relative to input DNA. The retrotransposons are grouped according to whether they show spreading for DNA methylation and H3K9me2, just H3K9me2 or neither of the marks. The profiles are shown for B73 leaf (A), Mo17 leaf (B), B73 endosperm (C) and B73 embryo (D). (TIF) [file pgen.1003127.s006.tif]

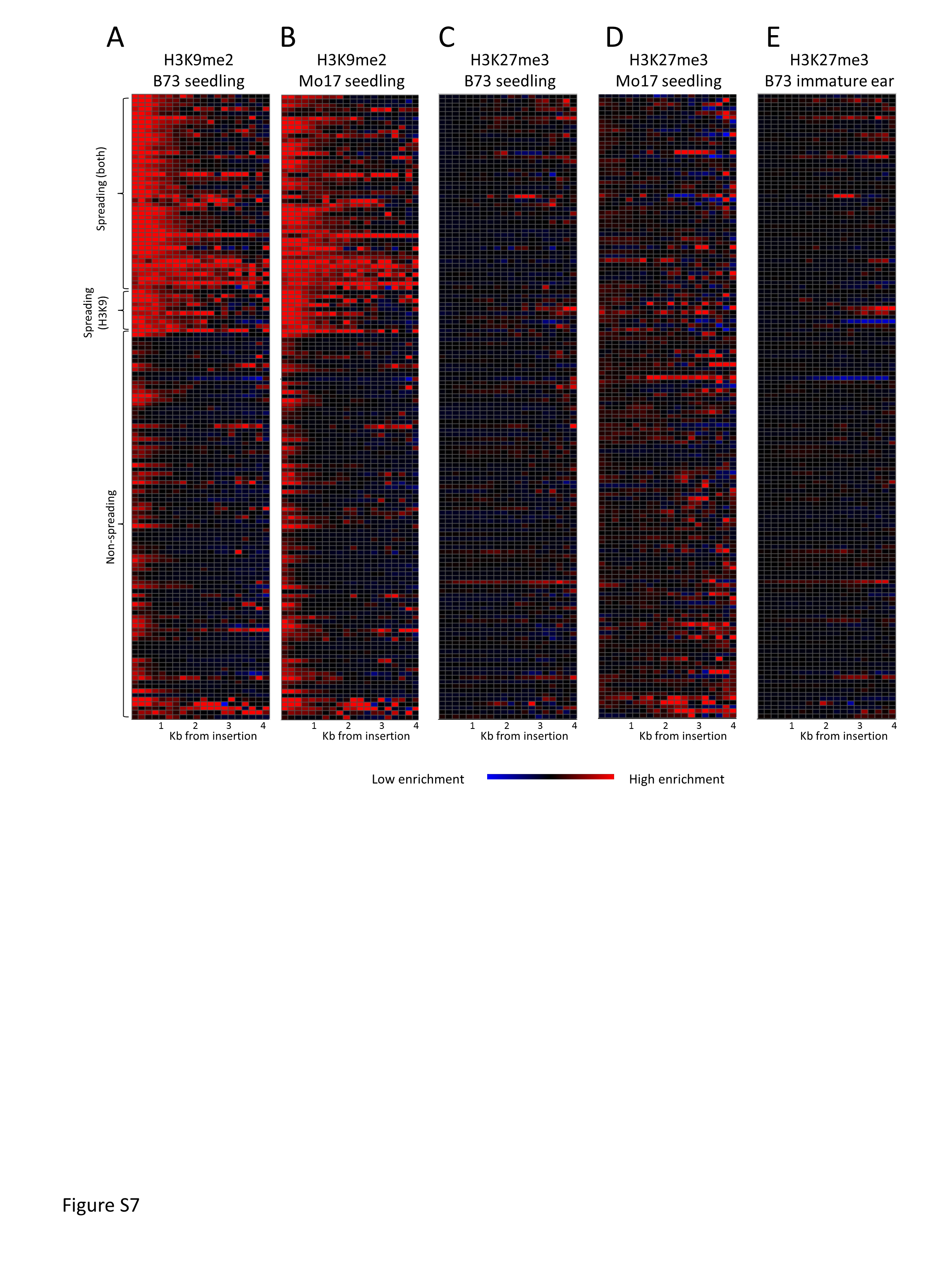

Supplement: Figure S7 — Histone modification patterns in different genotypes. The profile of several histone modifications is shown for both B73 and Mo17. The relative level of H3K9(di)- or H3K27(tri)-methylation in each 200 bp bin is shown for each of the 144 retrotransposon families. Modification levels calculated include probes flanking both ends of retrotransposable elements. The red color indicates enrichment for the modification while blue indicates depletion of the mark. Black indicates levels of the modification similar to genome-wide average values. The color intensity is based on the average log ratio of immunoprecipitated DNA relative to input DNA. The retrotransposons are grouped according to whether they show spreading for DNA methylation and H3K9me2, just H3K9me2 or neither of the marks. (A) and (B) display the profiles for H3K9me2 in B73 and Mo17 seedlings, respectively. (C) and (D) display the profiles for H3K27me3 in B73 and Mo17 seedlings, respectively. (E) shows the H3K27me3 profile for immature ear tissue from B73. (TIF) [file pgen.1003127.s007.tif]

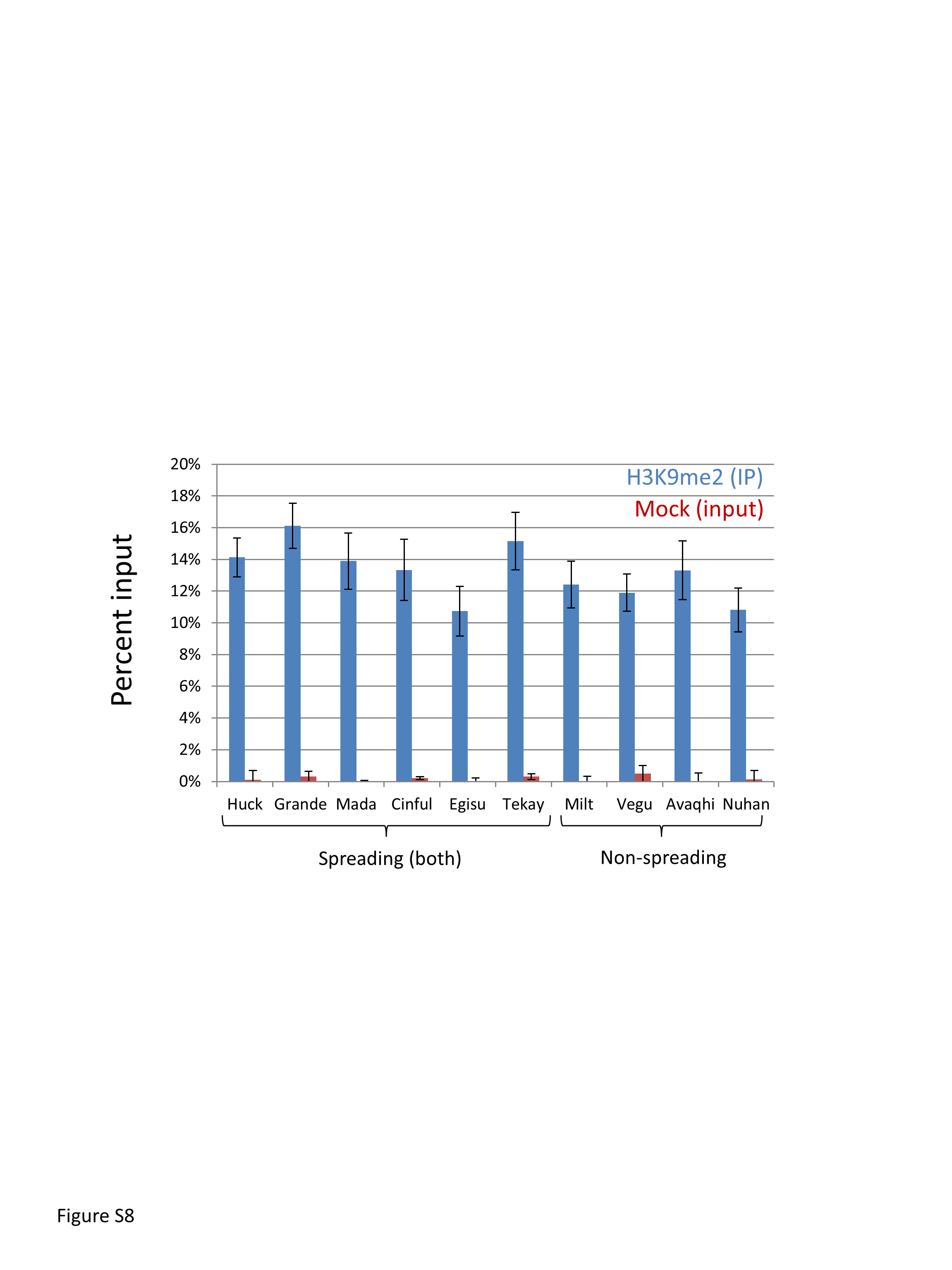

Supplement: Figure S8 — Similar levels of H3K9me2 within spreading and non-spreading retrotransposons. The ChIP-chip assay does not provide information on the abundance of H3K9me2 within repetitive regions. In order to assess whether the level of H3K9me2 was similar within retrotransposons with, and without, spreading we designed primers for internal sequences of 10 retrotransposon families including six with spreading (both) and four that were classified as non-spreading. The qPCR protocol described by Haring et al. (2007) was used to assess the percent input DNA recovered by immunoprecipitation. The percent of input DNA recovered after IP with the H3K9me2 antibody or a noIG control was determined for both sequences and the standard deviation is indicated with error bars. There were high levels of H3K9me2 in each of these retrotransposons but there was no significant difference between the spreading and non-spreading retrotransposons. (TIF) [file pgen.1003127.s008.tif]

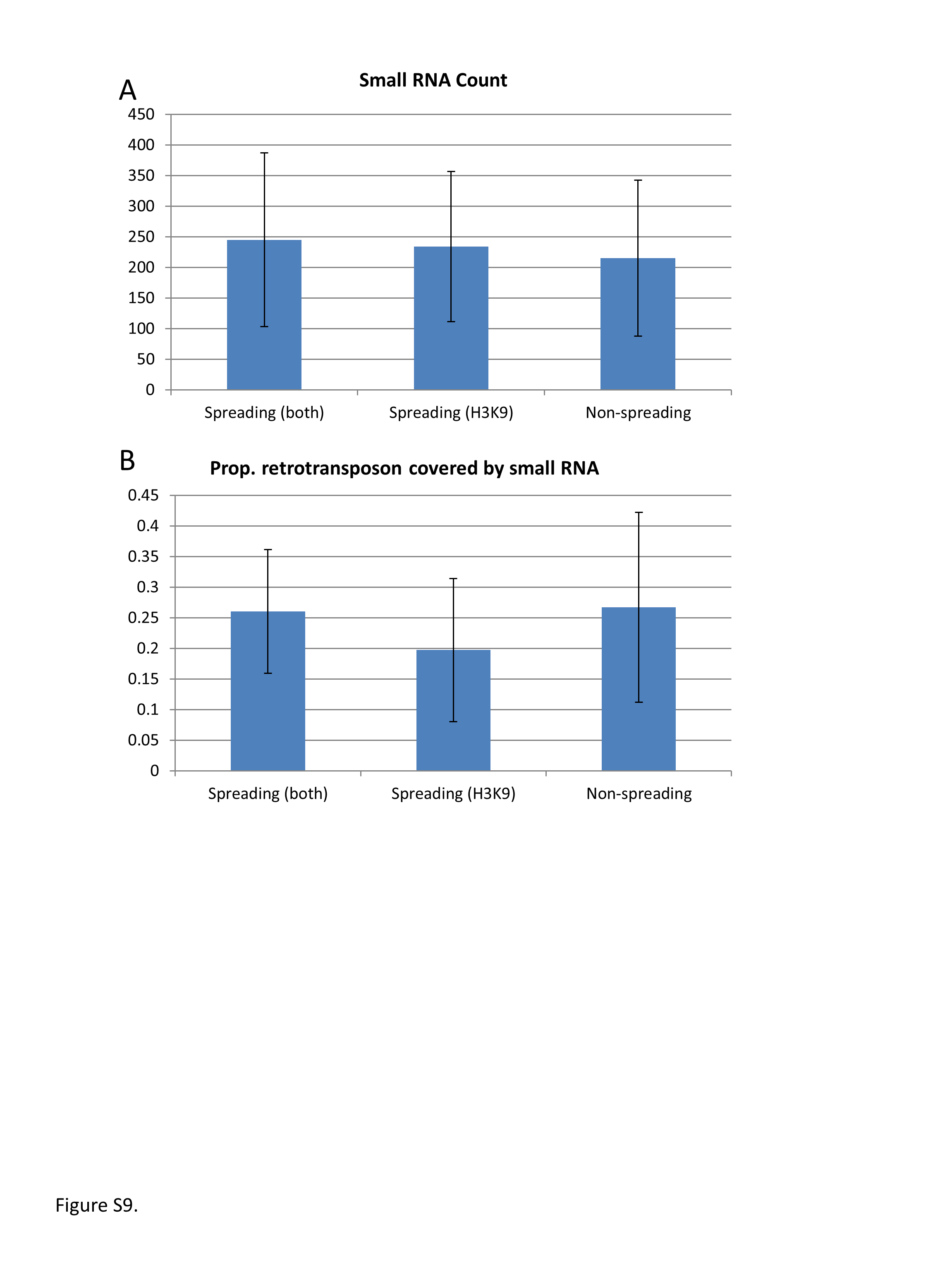

Supplement: Figure S9 — Small RNA coverage of retrotransposon families. B73 shoot small RNAs [47]. Small RNA reads for all size classes were mapped to the maize reference genome (AGPv2) using BLAT [63] under standard parameters. Coverage of small RNA reads over annotated maize transposons (ZmB73_5a) were calculated using BEDtools coverageBED [60]. (A) The average small RNA count per retrotransposon family was determined for each of the 144 retrotransposon families. The average count (and standard deviation) for families classified as spreading (both), spreading (H3K9) and non-spreading was then determined. There is no evidence for significant differences in the average small RNA count. (B) The proportion of each retrotransposon covered by small RNAs was then determined in a similar fashion. There is no significant difference in the proportion of coverage for the spreading and non-spreading families of retrotransposons. Small RNA data were downloaded from GEO as samples GSM918108 [47]. (TIF) [file pgen.1003127.s009.tif]

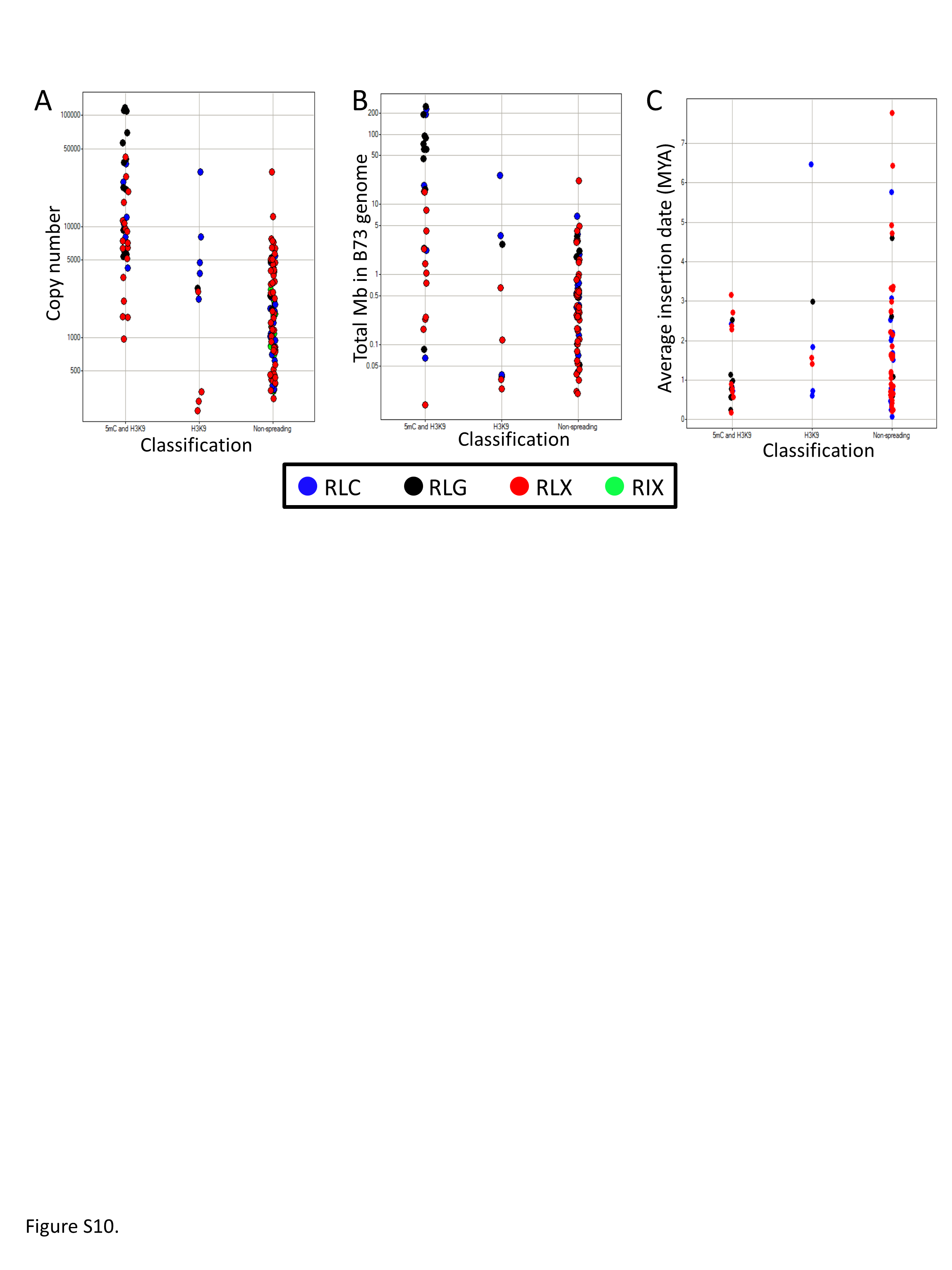

Supplement: Figure S10 — Characteristics of retrotransposon families with spreading of heterochromatic marks. In each of the plots the retrotransposon families are grouped into both (5 mC and H3K9), H3K9 only and non-spreading columns and the superfamily is indicated by the color. The data points are jittered to allow visualization of all families. (A) The genomic copy number of each family is shown using a log-scale. The families with spreading of both marks tend to have higher copy numbers. However, there is an overlap in the range of copy number for families with and without spreading. (B) The total Mb of the B73 genome comprised by each family is shown. (C) The average insertion date for each family is plotted. While the non-spreading families include both young and old retrotransposons the families with spreading of both marks tend to be younger families. (TIF) [file pgen.1003127.s010.tif]

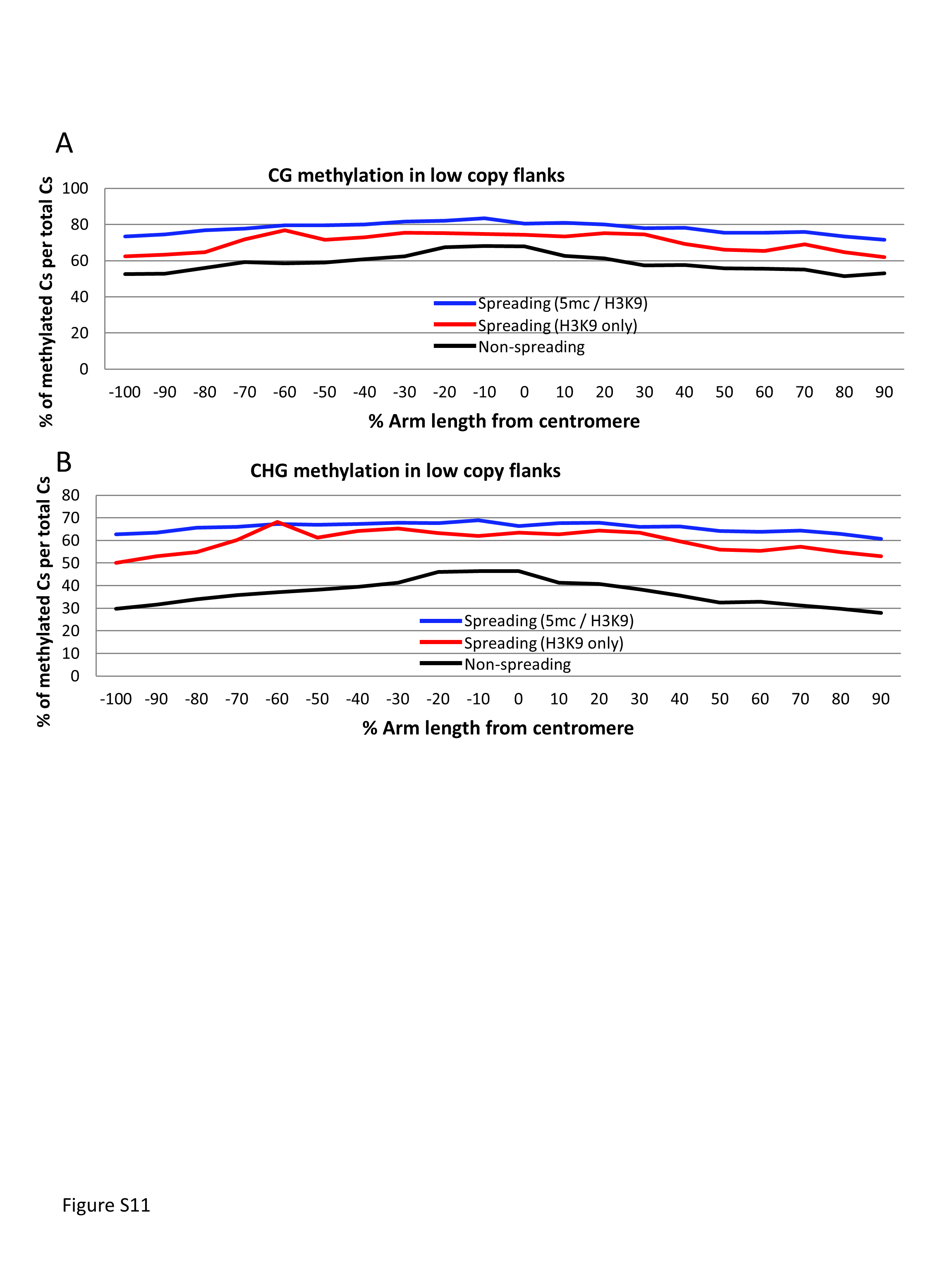

Supplement: Figure S11 — DNA methylation levels in flanking sequences are similar throughout the chromosome. The level of DNA methylation in sequences flanking retrotransposons was determined from bisulphite sequencing data. Only flanking sequences that did not contain any repetitive sequences within 1 kb of the retrotransposon were used. The proportion of methylated cytosines in CG (A) or CHG (B) contexts was determined for 1 kb of low copy sequences flanking spreading (both), spreading (H3K9) and non-spreading retrotransposon families. (TIF) [file pgen.1003127.s011.tif]

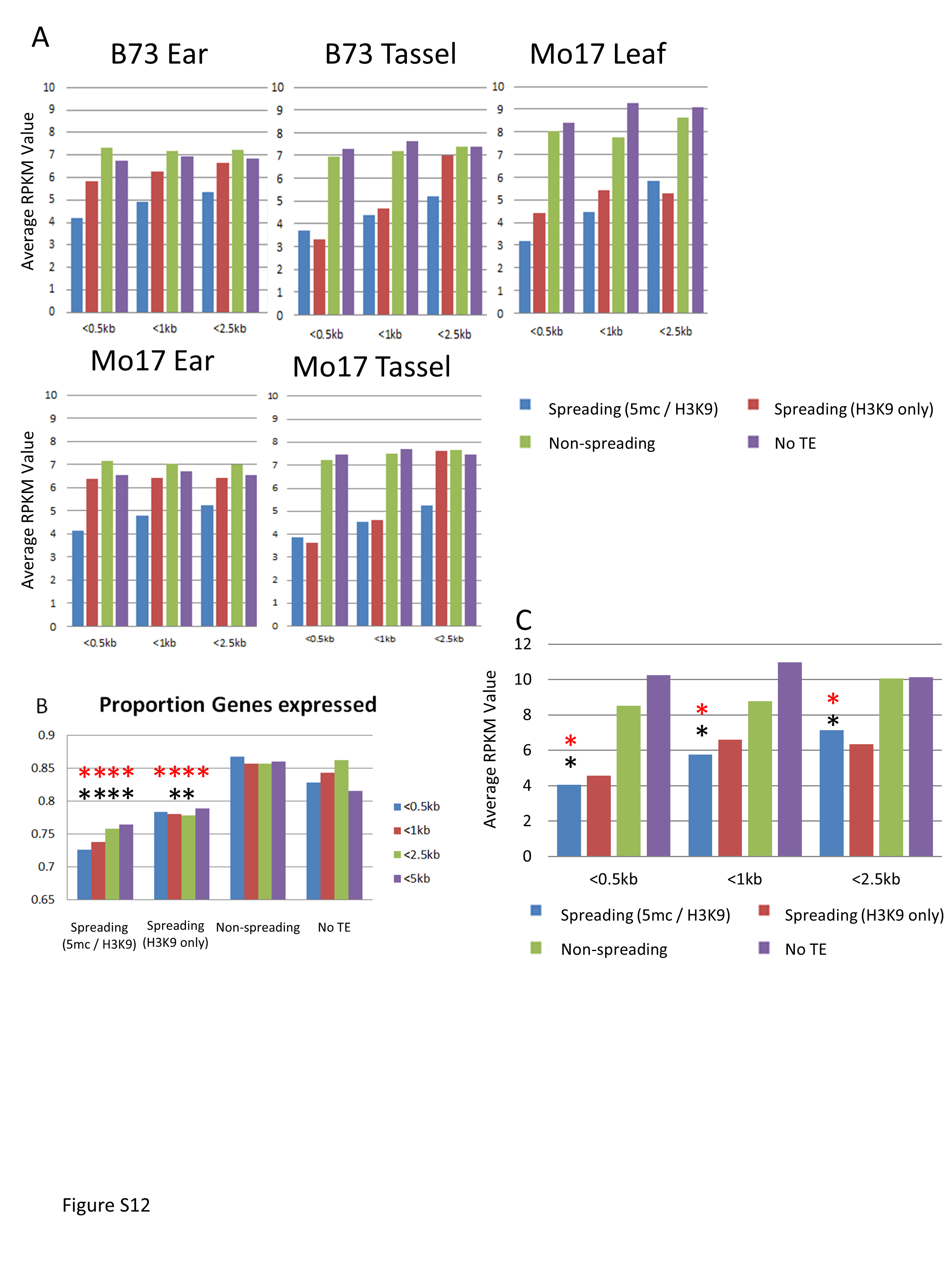

Supplement: Figure S12 — Genes near spreading retrotransposons show lower expression than genes near non-spreading retrotransposons. (A) Average RPKM values (from B73 Ear, B73 Tassel, Mo17 leaf, Mo17 Ear, and Mo17 Tassel tissues) for all genes falling within 0.5 kb, 1 kb, and 2.5 kb from the respective class of retrotransposons were determined. (B) Proportion of genes expressed (RPKM>0) in B73 leaf tissue for genes near 5 mc and H3K9 spreading elements, H3k9 spreading only, non-spreading, and no TE nearby. Red asterisks indicate highly significant (p<0.001) difference from the non-spreading elements within the distance classification. Black asterisks indicate significant (p<0.001) difference from genes not near any TE. (C) Average RPKM values in B73 leaf tissue for expressed genes (all genes with RPKM = 0 were omitted) falling within 0.5 kb, 1 kb, and 2.5 kb from the respective class of retrotransposons were developed. Red asterisks indicate significant (p<0.05) difference from the non-spreading elements (green) within the distance classification. Black asterisks indicate significant (p<0.05) difference from genes not near any retrotransposons (purple). (TIF) [file pgen.1003127.s012.tif]
